# Bibliographical Notices

**Published:** 1844-07-01

**Authors:** 


### Periscope;

OR,

### CIRCUMSPECTIVE REVIEW.

" Ore trahit quodcunque potest, atque addit acervo."

### Notices of some New Works.

Mesmerism and its Opponents. By *George Sandby*, jun. M.A. Vicar of Flixton, Suffolk. Longman and Co. 1844.

We cannot but think that the practice of Mesmerism is a very questionable *vicarious* employment, for that of teaching the doctrines of Christianity to the good folks of Flixton, in Suffolk. We should think that Mr. Sandby would do much more good by praying with the sick of his vicarage than by pawing them over on the bed of suffering or death, and distracting their minds from the awful preparation for another world. Is there no bishop to watch the conduct of the Suffolk clergy? Mr. Sandby looks forward, of course, to be victimised, like Galileo, by the Pope; but in this he will be disappointed. He will, we imagine, receive a quiet hint from his superior in the church, at home, to lay aside the mummeries of Mesmerism, and attend to the spiritual wants of his flock. We shall not take the trouble of wading through the trash of which this volume is composed—hashed and raked up from old tales and cases that have been over and over again proved to be falsehoods and deceptions. Thus, the Deptford imposition is recorded once more, as though it were true as Holy Writ, while every one knows that it was a piece of as glaring mendacity as even the annals of Mesmerism can produce!

The Vicar of Flixton tells us that belief in the truth of Mesmerism has now spread the whole length and breadth of Great Britain—excepting among the great bulk of the medical profession! And why has it not taken root there? The reader will be a little surprised at the following reason for medical scepticism.

" But if Mr. Wakley did not succeed in disproving the honesty of two excellent sisters, there was one thing in which he was eminently fortunate. The thunders of the 'Lancet' had their intended effect on his medical brethren. Though anything but a favourite with them before, he henceforward became their pet authority. And strange to say he also became their terror. Fearful of being hitched into a line of the next week's 'Lancet,' as believers in the so-called absurdity, some gentlemen straightway swallowed their rising convictions with wry faces and reluctant hearts;—while the remainder, *almost to a man*, refused for the future to be present at any Mesmeric demonstration whatsoever. Like Mr. M'Neile at Liverpool, they carefully retreated from the evidence of their own senses, but from a different reason altogether. My clerical brother judged that there was something supernatural in these cases;—he regretted that he had not faith to play the part of exorciser and bid the devil depart, and from want of this faith would '*see nothing of it.*' But the fears of the liberal profession were of a different order. It was not of an evil spirit that *they* stood in awe; it was of Mr. Wakley,—of the evil genius of the 'Lancet.' "

So the medical profession, " almost to a man," refuses to believe in Mesmerism, because Mr. Wakley detected the impostures of the Misses Okey!

On former occasions, we stated that, if Mesmerism were a reality, there would be infinite danger from it—that no man's life or woman's virtue would be safe. But we said at the time, that there was no such danger, because Mesmerism was a gross humbug. The Vicar, however, believing in the truth of Mesmerism, is forced to admit that "there are certain dangers touching *la morale.*" And how does he get over these?

"If it is meant that under the *pretext* of Mesmerising, in a case where Mesmerism is not required, parties can avail themselves of the occasion to commit an offence *contres les bonnes mœurs,* I am not careful to enter upon the objection. *Men sometimes go to church from the most improper motives;* men sometimes read the Scriptures with no other view than that of finding food for ribaldry and unbelief; still, as has been often said, who would shut up our churches or burn our Bibles on that account? Again, we say the abuse of a thing proves nothing against its value. If parties, in sport or in thoughtlessness, throw themselves into the power of an unprincipled acquaintance, with them lies the fault, and they must take the consequences. Still I have my doubts whether Mesmerism does afford the easy opening for misconduct, with which it has been taxed. The deep sleep or torpor which would place the sleeper so completely at the mercy of the Mesmeriser, as to give an opportunity for evil, does not occur every day;—and more generally, *if not always,* the Mesmeric state produces, on the part of the patients, *such a high tone of spirituality and sense of right,* as to make them less than ever disposed to an acquiescence in what is wrong."

So, then, the soporific effects of a dull sermon, and a stupid preacher, are to be put into comparison with that Mesmeric condition in which a man or a woman may have their legs cut off without the slightest consciousness of the operation!! Oh, admirable reasoning!

The Vicar's chapter on the contrast or comparison of the Miracles of Christ with those of Mesmerism, had better been omitted. We maintain that they are not precisely identical, and, therefore, they must be totally different. Mesmerism, he avers, does not pretend to turn water into wine, or to walk on the sea. No. But if it enable a girl to see through her navel as far as China or Peru, we maintain that this is, if possible, the greater miracle of the two! We are not, indeed, among the bigots who would throw the Vicar of Flixton into a dungeon, for instituting such odious comparisons on such sacred subjects; but we certainly would recommend his bishop to give the vicar a hint to mind his own business—"*ne sutor ultra crepidam.*" We shall dismiss the subject with the following short extract.

"Another objection is, that the sleeper is placed in an undesirable state of feeling in regard to the Mesmeriser; that there is an attraction towards him,—something amounting to affection, or even love; and that this state of mind or feeling reduces the patient to an improper dependence on the will of another. That, in the Mesmeric state, the sympathy between the Mesmeriser and the sleeper is powerful and extraordinary, we all know; it is one of the most curious phenomena. The sensibility that is then produced, is singular in the extreme. But the feeling is rather that which exists between two sisters than any thing else; it is a feeling which has regard to the happiness, and the state of moral being of the Mesmeriser; which is alive to injuries or pain inflicted on him,—which *desires his well-being here and hereafter.*"

The VICAR tells us that the whole of the medical profession is deterred from embracing MESMERISM by the rod of Mr. Wakley! We wish there was a coroner on the bench of bishops to exercise a similar and salutary restraint on the clergy of this country.

---

MEDICINES, THEIR USES AND MODE OF ADMINISTRATION; INCLUDING A COMPLETE CONSPECTUS OF THE THREE BRITISH PHARMACOPŒIAS, AN ACCOUNT OF ALL THE NEW REMEDIES, AND AN APPENDIX OF FORMULÆ. By *J. Moore Neligan,* M.D. Dublin, London, 1844.

THE object avowed by the author in the compilation of this book is, to furnish a concise view, but as complete as possible, of the different substances, both simple and compound, which are deemed worthy of a place in the Materia Medica. In the arrangement of the articles of the Materia Medica he has adopted a physiological classification, from a conviction of "the great practical value of an arrangement, which is based on the ultimate medicinal effects of remediate agents." Were we disposed to be hypercritical, we might object to the author's saying that a physiological classification is one "based on the ULTIMATE medicinal effects of remediate agents." A physiological classification is one based on the IMMEDIATE, or as they are sometimes called, *primary* effects of medicinal agents. The absence of the natural and chemical history of the Materia Medica renders the work an inadequate substitute for a Dispensatory; indeed, we are strongly inclined to think, that the same reasons which induced Dr. Paris to omit the department of Special Pharmacology from his recent Edition of the Pharmacologia, should have prevented Dr. Neligan from sending his work to press. The table of contents is very large indeed, extending to something more than twenty-four pages; this facilitates reference very considerably, a matter of no little importance in a work of this kind, where the arrangement adopted is of so unusual a description. The several classes are arranged alphabetically, and are as follows (each class occupies a Chapter). 1. Antacids. 2. Anthelmintics. 3. Antispasmodics. 4. Astringents. 5. Cathartics. 6. Caustics. 7. Diaphoretics. 8. Diuretics. 9. Emetics. 10. Emmenagogues. 11. Emollients. 12. Epispastics. 13. Errhines. 14. Expectorants. 15. Narcotics. 16. Refrigerants. 17. Sedatives. 18. Sialogogues. 19. Stimulants (general). 20. Stimulants (special). 21. Tonics. The last or twenty-second Chapter contains what the author calls "supplementary agents;" under this head are included the different articles which, though not employed in medicine for their remediate powers, are in frequent use as colouring agents, perfumes, tests, and pharmaceutical agents. Next comes an Appendix of Formulæ, which is however very restricted, being confined principally to the new remedies described in the work, or to those not yet in general use. This is followed by a posological table; now the doses of the various medicines being invariably given throughout the body of the work to which such tables are appended, we have always thought that these posological tables were of comparatively little use, and may therefore be very well omitted; they are in fact mere filling stuff. Truth obliges us to say that, after running over Dr. Neligan's book, we have been unable to find any thing in it of that novel or original character, such as to entitle the author to any encomiums from us as one who has contributed to the extension of medical knowledge. The work is one of mere compilation, containing nothing whatever that can render it likely to supersede any of the very numerous works already published on the same subject. The descriptions are far too meagre to allow the student to dispense with the Dispensatory or Dictionary of Materia Medica. In fact, the book is little more than a large conspectus of the three Pharmacopœias of London, Dublin and Edinburgh. The alphabetical mode of arranging the several classes of medicines we do not like. We consider Old Murray's much more rational; it is the one indeed which forms the basis of the arrangements that have been adopted by subsequent writers on Pharmacology in this country. It is not a little repulsive to our ordinary modes of thinking on these matters to find Caustics immediately following Cathartics, Epispastics following immediately on the heels of Emollients. We never met a classification which so decidedly sets at defiance the laws of good division, as the one adopted by Dr. Neligan. Still the book is one which we have no doubt will prove serviceable to the student and junior practitioner, for whose especial use the author seems to have intended it. Considerable care has evidently been bestowed on its compilation.

On the Absorption of Blood and Pus. By *Dr. Zimmermann.* (Condensed from the Beiträge zur Physiologischen und Pathologischen Chemie und Mikroskopie, &c. Berlin, 1843.)

If the theory of Absorption, so far as relates to the absorption of fluid substances in a purely physical way (endosmosis and exosmosis), was already beset with its own very serious difficulties, this was still more the case with fluids which contained solid molecules, as, for instance, chyle, blood, pus, &c. so that to those pathologists, who for several years invariably followed in the footsteps of the physiologists, the absorption of pus was absolutely impossible, and they were forced to have recourse to hypothetical modes of accounting for the circumstance, wherein the vis medicatrix naturæ performed a very prominent part.

That pus cannot be absorbed as pus from abscesses, serous cavities, &c. so long as the blood-vessels, and lymphatic vessels are completely uninjured in their structure, is at the present day admitted on all hands, and for this advance in the knowledge and investigation of the difficult subject regarding pus-absorption we are indebted to a strict micrometrical examination of pus-globules and of the capillary vessels. An absorption either of the blood-vesicles or of the pus-globules in the way in which homogeneous fluids are absorbed, cannot be maintained, and all the arguments advanced in defence of it rest either on erroneous hypotheses, or on false conclusions. Dr. Piorry, for instance, in his recent paper on pyemia states, that a resorption of pus-corpuscles may take place, whilst the blood-vessels and lymphatic vessels remain uninjured, because blood-vesicles, which, according to Milne Edwards, differ but little from the former in size, have been re-absorbed, as in ecchymoses, for example; in answer to this it may be stated, in the first place, that the pus-corpuscles are in general not so large as the blood-vesicles, and secondly, that their resorption in cases of ecchymosis may by no means follow, as Dr. Piorry supposes. In the same way we may dispose of another argument advanced by him, namely, that just as well as the pus-corpuscles make their exit from the uninjured vessels, these same pus-corpuscles may be re-absorbed again.

For an escape of blood-vesicles or of pus-corpuscles from the blood-vessels can only occur in one of two ways: either the vessel is torn through excessive expansion occasioned by an accumulation of blood or pus, or, as frequently seems to happen with respect to the latter, the cells of the vessel have been metamorphosed into pus by contact: the vessel thus becomes broken in its continuity, it is no longer uninjured, even though the aperture exhibited by it should not be larger than the diameter of a single pus-corpuscle. Who, for instance, would assert, if a very fine needle were swallowed, and if this comes out at the perinæum, that it made its way from the stomach to the intestine, without having destroyed the continuity of the tissues through which it passed? the same happens with respect to a pus-corpuscle. So that the smallness of the aperture can make no difference.

If blood-vesicles, together with the other constituents of the blood, are capable of passing through the parietes of the capillary vessels in this way, it does not appear probable that they perforate the parietes of these individually: it may be that, in consequence of the active or passive state of congestion, an actual laceration of the finer blood-vessels takes place, which occurs so much the more easily, if the vessels have room for distention, as, for example, in the brain, or in the membranes, when the epithelial covering is detached from these, as in consequence of a vesicatory.

The best idea as to the manner in which the absorption of the blood-vesicles may occur, we derive from ecchymoses occasioned by external violence. Here the fine capillary vessels are crushed together with the blood-vesicles circulating in them, and into the interstices of the cellular tissue there is poured out still more blood, which has to run through a cycle of changes, before it is re-absorbed. For we remark that the place affected by the external violence first becomes red and blue, then blue, then blue-green, green-yellow, and finally yellow; then this colour disappears gradually, and the cellular tissue again assumes the normal appearance. It is manifest that these changes of colour can be produced only by chemical changes of the hematin, and we have here almost the same process before our eyes, as that which the blood-vesicles probably undergo through the power of the hepatic cells on the gall-ducts. On the contrary, this series of changes, which the red colouring matter of the blood undergoes in those parts of the skin which have been contused, I have not at all been able to observe in the case of petechiæ, where the blue red colour passes gradually into the clear red, the presence of which might be observed probably after disease has lasted for two months. In both these facts there seems to be contained a proof, that the blood-vesicles are not re-absorbed as blood-vesicles: in the former case they, together with the other parts that have been injured, follow evidently chemico-organic laws, if they have run through the series of changes, if the capillary vascular net-work has been again re-established; the cells of the same exercise on the metamorphosed constituents of the blood, the influence, which we cannot describe, but the absorption of which we observe as a consequence of it.

In actual extravasations of blood occasioned by injury of large blood-vessels, and where blood is effused in considerable quantities into cavities, matters are arranged somewhat differently. For here, because the blood has now lost those conditions which kept it fluid, a longer or shorter time after it has been effused, coagulation takes place, in consequence of which, a separation of the blood into its three natural constituents takes place. The process of absorption can be most easily accounted for, where the blood was effused into cavities lined by serous or mucous membranes, whilst the accounting for the thing is more difficult in cases where, by reason of an inflammatory process excited by the extravasation, a capsule resembling a mucous membrane, must first form around the coagulated blood, the cells of which capsule, together with those of its blood-vessels, effect absorption by their peculiar power. Since other cases do not occur to me just at this moment, I shall quote one, which fell under my observation, and which satisfied me that the resorption of the serum first commences after coagulation. The case was that of a soldier who, after an injury of the knee, had a considerable quantity of blood extravasated in the synovial capsule above the patella. As the nature of the swelling hereby occasioned could not be accurately ascertained, an attempt was made to discuss this swelling, and to promote the absorption of its contents, whether pus, liquor sanguinis, &c. by means of fomentations, solution of muriate of ammonia, and afterwards by suitable pressure. When, however, after the lapse of eleven days, the tense and elastic tumor, which however presented no redness whatever, had been but very slightly diminished, it was opened. The pitchy black blood which issued from the opening was coagulated in clots, and after standing for four hours yielded no serum. The coagulum, which amounted to about six drachms, contained a considerable quantity of fibrine, which, after pressing the coagulum through a linen cloth, remained behind as a short-grained crummy, red coloured mass, and did not become thoroughly white, even after lying for a considerable time in water. The cruor, when well dried, yielded, from 1000 grains, 346 grains of solid substance, which was quite black, and did not exhibit a shining fracture, as blood not deprived of its serum, and well dried, usually does. The great quantity of solid substance, obtained from the cruor, which exceeds that from ordinary blood by about 140 grains, proves to me that within these eleven days the absorption of the serum had almost been completely terminated, and the blood-clot contained only coagulated fibrine and blood-vesicles. This case was brought forward in order to afford a practical proof against the purely theoretical views of those who maintain that blood extravasated within the cavities of the human body cannot coagulate. The views opposed to mine have been recently defended by Steifensand in his treatise on nerve and blood, which was written in order to mediate between the extremes of the Humoral and Solid Pathology; but the grounds on which his arguments rest, have been already proved false by others. In order to show on how weak a footing the arguments of the adversaries rest, I shall merely state that the sanguineous tumors in the heads of new-born infants, in which cases the blood is seldom or never found coagulated, can afford no kind of proof whatever. For the blood of new-born infants contains, as has been clearly shewn, either very little or no fibrine whatever, which alone can produce coagulation. The same thing occurs in the case of sanguineous apoplexy, where the blood is not found coagulated even after a considerable time; for it is known that in 1000 grains of the blood of those affected with sanguineous apoplexy there are not as much as 2 grains of dried, or from 5 to 7 of moist fibrine present. And the case just now instanced clearly shews, that absorption does not commence with the fibrine, but with the albumen, and in general with the constituents of the blood soluble in serum. The principal reason why this takes place is, that fluids are absorbed sooner than solid parts, and it cannot be denied that the process of Endosmosis and Exosmosis must not be disregarded in the present case. And how this process may be accelerated by continued pressure of the many and valuable effects of which modern surgery has carefully availed itself, may be readily explained according to physical laws, if these could be made to account for vital actions, such as Absorption is.

With respect to the changes which the blood-vesicles of the coagulated blood deprived of their original properties run through, whether such changes take place according to the laws inherent in every organic combination or under the influence of the cells of the synovial membrane surrounding them, and their blood-vessels, whether they occur in the same way, as may be observed in the case of superficial ecchymosis, so as to become changed into matters resembling the constituents of the bile, and thus be rendered capable of being absorbed; who could ascertain all this, and what facts have been collected hitherto having a bearing on the subject? To explain how the coagulated fibrin is re-absorbed appears to me extremely difficult; it is either not taken up at all, or not till a very late period, and affords material for those indurations and concretions that remain behind, such as are observed after these extravasations. I consider it not undeserving of attention that the constituents of the extravasated blood, as for instance its plasma, evince no tendency to organization, a thing which takes place readily under other conditions, where the fibrin is not in a mass, but is either coagulated in the molecular form, or runs through a series of changes, as the exsudation-corpuscles, around which cells form, or shew its change into true pus. The presence of the blood-vesicles must in every case exercise a peculiar influence on the coagulation of fibrin, as we know that pure plasma without the body coagulates but very slowly. It seems probable for many reasons that the constituents of extravasated blood, in case they become re-absorbed, are no longer capable of keeping up the so-called progressive changes (formation of cells) as pathologists generally admit, but that they, entering the circulation, run through a cycle of organico-chemical changes as well through the action of oxygen, as of the changing power of the cells in the excreting organs, the ultimate products of which seem to be the constituents of the urine, bile, and sweat. This however depends in a great measure on the time during which the extravasated blood remained without the vascular system, before it was re-absorbed; the longer it remained so, the more depressed did the inherent powers in the blood-cells become, the more the vitality inherent in them, and the organic power of elective affinity which is in general inherent in the organic atoms yield to the influence of chemical laws, so much the less are they in a state, to be again susceptible of vitality in the mass, and so much the less capable are they of imparting this vitality, a thing which is necessary for the progressive changes. As an instance of the contrary, the case may be adduced, where blood, after being deprived of its fibrin, being injected into the veins of a bloodless animal, re-animated this animal anew, but here the case is different, as may be readily seen.

With respect now to the absorption of pus, in accounting for which the medical practitioner is so deeply interested, I am inclined to think that we must admit that there is more than one way in which this may take place. In fact, the ideas regarding pus-absorption, and with respect to what becomes of the pus when absorbed, would not be so contradictory and obscure, if pathologists had taken the trouble, at the time the pus became absorbed from an abscess, to examine the blood as well as the excretions of the patient. The absorption of pus may, in my opinion, take place in two ways; firstly, as real pus, that is, the pus-corpuscles are taken up, just as they are, by the vascular system, and secondly, the pus, before it is re-absorbed, undergoes certain changes, which render it more fitted as well for absorption, as for further elimination by the excretory organs. For both cases, pathological anatomy, as well as pathological chemistry, have collected numerous instances and observations.

With respect to the first mode, no one can deny, that the vessels can be in a perfect state of integrity. Either from the very onset, through the stasis which preceded the exudation or extravasation of the plasma, from which the pus was subsequently formed, a rupture of some vessel took place, and this continued so long, and was still further increased through the so-called dissolving power of the pus, or through the latter circumstance alone, the vessels touched by the pus were injured in their continuity and presented to the corpuscles an aperture, which could readily afford them a passage. This dissolving power of the pus, that is, its power to dispose for the same changes, which itself had undergone, the cells of several structures, which in their chemical properties resemble albumen and fibrin, this powver, I say, is very similar to contagion, if not altogether identical with it. But it is by no means sufficient to have shewn how, for instance, in abscesses the first step to its absorption took place by the action of the pus itself: for, notwithstanding the opening of the vascular terminations, the pus-corpuscles would have remained quietly in their place, had not certain influences occasioned their admission into the vascular system. One of these is the pressure made by the parts inclosing the pus; for we may often observe, that the absorption of the pus goes on best, when the purulent tumor has attained its highest stage. In order to promote the absorption of pus, surgery often employs several means, which effect either a pressure, or indirectly an energetic contraction of the parietes of the abscess. Cataplasms, besides their other effects, act in the former way. In this way, we may in part account for the effects of a treatment, which, instead of opening large abscesses, consists in touching their external covering in different places, either with corrosive substances, or with the cautery. It is evident that, by the subsequent inflammation in the external integuments, a considerable tumor is produced, and accordingly pressure is made on the pus. The other influence which contributes to the admission of pus-corpuscles into the vascular system, must be in every case the suction-power of the heart, which is so very striking on the venous system. In consequence of the stream of the circulating blood in the veins having its draw towards the heart, the pus-corpuscles, which are at the orifice, and by the vessel itself, are hurried along and carried into the circulation. This is precisely the same as when at the resolution of the stasis in the vessels the circulation begins again, which in like manner must be in a great measure set down to the account of the suction-power of the heart.

The other way in which pus may be absorbed, is as follows. I have observed in certain patients, that at the time when an absorption of pus took place, as it seemed, the urine of these patients was coagulable, and was indebted for this quality to the fibrin dissolved in the urine. In the case of two patients, of whom the one had pleuritis, the other pneumonia, I found a serum containing fibrin in the former at the third, in the latter at the second bleeding, whilst the blood of the first bleeding presented that of the genuine inflammatory blood, and just at the same time I found the urine of both these patients coagulable from containing fibrin. Besides, I satisfied myself that this urine contained no pus-corpuscles whatever. In consequence of this, I suspected that probably the pus-corpuscles had broken down about this time by some means or other, if not into the fluid plasma from which they had been originally formed, into a quantity of small fragments or molecules, the absorption of which by the open ends of the fine capillary vessels was now easily effected. In what way and by the combination of what circumstances this breaking down of the pus-corpuscles and their re-formation into fibrin takes place, I cannot say; it is well known that acetic acid on touching pus-corpuscles, cause them to break down into small kernels; at the time I remarked the fibrinous urine, and this happened at a time when the abscess had attained its highest degree of maturity, could any acid have become developed, as lactic acid which occasioned the breaking down of the pus-corpuscles? or did this breaking down take place spontaneously by reason of the pus-corpuscles tarrying for so long a time in the abscess? or was it occasioned through the influence of the membrane lining the abscess? who could ascertain this? I have already mentioned a mode of treatment employed in surgery in order to promote the absorption of pus, by corrosives and burning. The effect of this method is frequently astonishing, and I think that, by the action of the same process, this breaking down of the pus-corpuscles is produced. It is to be regretted, that in the case of such patients medical men have never concerned themselves, as to what became of the absorbed pus; they were merely astonished at it, muttered something of the vis medicatrix naturæ, and those who thought of the matter were of opinion that the absorbed pus, as it still contained constituents of blood, became again changed into normal blood, and was fitted for nutrition. Accordingly they advised not to open very large abscesses, but first to treat them in the way already mentioned, and subsequently, after a great portion of the pus became absorbed, to let the other portion be discharged.

I now come to the second question, viz. What becomes of the pus that has been absorbed, and is now circulating, or of the fibrin existing in the blood in the molecular form? Before I enter more closely into this question, I must first recur to the traditional views entertained by those who think that this absorbed pus may again become changed into the normal constituents of the blood, and become fit for nutrition. My grounds are as follows. In the first place, we must admit that the plasma, from which the pus was subsequently formed, was exsuded only for this reason, because, by its abnormal properties, whether in respect of quality or quantity or both, it so irritated the organic nerves and capillary system, together with the cells of a tissue, that from some cause or other it became converted into a locus minoris resistentiæ, so that a stasis and deposition of pseudo-plasma ensued. Accordingly, by a series of physiologico-pathological processes, the mass of blood was freed from a part of its constituents, which had now become abnormal, and which endangered its entire mass.*

---

* The qualitative differences of the fibrin in the inflammatory blood and of that existing in healthy blood, are observable partly through its physical, partly through its chemical, characters. With respect to the latter, there is to be found in Casper's Wochenschrift (1843. Nr. 42) a remark by Professor Mulder to the following purport. " I have found in the blood a matter which is formed from the fibrin, and may be considered as oxidized fibrin. This is found in great quantity in the blood in inflammatory fevers, and, in fact, inflammation shews itself by a greater quantity of oxidized fibrin, properly speaking, Proteine. The old idea of inflammation is therefore confirmed. This substance may be presented

If now this pseudo-plasma, at the time it was exsuded, was so defective in its quality, as not to be fit for its destination, namely, for nutrition (cell-formation), how much more truly this may be said of it, after it had run through a series of other changes. The two normal constituents of the plasma are the albuminate of soda (Natron-Albuminat), and the fibrine, with fat and some salts; we have, for instance, to consider fibrin, whose quantity as well as quality appears changed, as the proper materia peccans, whilst the albuminate of soda is probably not so, and is exsuded with it merely for this reason, because with the fibrin it forms the fluid and most readily transuding constituent of the blood. Therefore, it seems very probable that the albuminate of soda may be re-absorbed, and be further subservient to the progressive changes, for which it seems to be peculiarly intended,* if absorption take place early. The fibrin, on the contrary, which is soon coagulated in the molecular form, and chiefly forms the pus-corpuscles, resists absorption on this account, and if it is ultimately again taken up by the mass of blood, it must be in a state to become subservient to those progressive changes, as it is in a great measure eliminated by the excretory organs. By a series of facts, which have been accumulated by a variety of observers from time to time, regarding the origin, nature, and importance of the fibrine in the blood, I have arrived at a mode of thinking widely differing from that usually adopted; the final result of my investigations on this point is this: that the fibrin owes its origin to the regressive cell-changes of the muscular structure, and accordingly exists in the blood probably as a purely excrementitious matter. This view is countenanced by my observation, that at the time when the absorption of the effused plasma or pus was going on, the serum of the blood of the two pulmonary patients just mentioned contained the absorbed fibrin in the molecular form. This fibrin-containing serum presented itself at the same time when these patients voided urine which contained fibrin, and certainly it is not going too far to conclude that both phenomena stand to each other in a very decided relation with respect to their cause. If then, I ask, the fibrin is destined, according to the views of almost all physiologists, as the most highly organized Protein-combination for cell-formation, why should it be eliminated by the kidneys, as well in its natural form, as in various other forms of metamorphosis?†

After having endeavoured to shew that probably the albuminated soda absorbed from an abscess, but not the fibrine absorbed in the form of pus-corpuscles or small solid molecules, may again become available for the purposes of

---

in various ways, and most readily by boiling fibrine or albumen for several hours under the influence of the air. In healthy blood there is but little of this present. It is prepared in the lungs. The red colouring matter of the blood is accordingly not only the carrier of oxygen, but also of fibrine." [See in the preceding number of the Medico-Chirurg. Review, Prof. Mulder on the Oxides of Protein, where this subject is treated more in detail.—REV.]

* This is further corroborated by the circumstance, that it was not albumen but fibrin I found in the urine of the patients at the time absorption took place. If the re-absorbed albumen had not been more fitted for the progressive changes, it would probably have been eliminated by the kidneys, as well as the fibrin.

† Von WALTHER (System der Chirurgie, 1843) is of opinion, that absorbed pus is no longer capable of nutrition. He says, (p. 85), "Pus is absorbed, not however, as such and unchanged—by the veins, traverses the mass of blood, acting prejudicially on it, to the depuratory organs; in the latter it may be separated from the blood, as a substance no longer formative, becomes mixed with the excretory fluids, and is eliminated with them"—and again, (p. 80), "all formative, recrementitious, animal fluid, (chyle, lymph, blood) are coagulable. No excrenutrition, it still remains for me to point out the two ways in which the pus or its fibrinous constituents are further disposed of.

The first case is, that either the absorbed pus-corpuscles or the molecular fibrine are deposited in another organ, and there form a new abscess. This occurrence, which is rather frequent, was easily accounted for, as Nature herself shewed that, between the first and second abscess, or the disease occasioned thereby, a causal connexion existed. If we admit that, by whatever causes it may take place, the quantitative and qualitative plasma was at first withdrawn, we must consider the first deposition of the same and the formation of abscess, which I shall call metastases of the first grade, as a critical process, which was commenced and perfected through the pseudo-plasma and the existence of a locus minoris resistentiæ. For the blood itself was thereby partly freed from the anomalous constituent. If the pus-corpuscles absorbed from the abscess were again deposited in any organ, this is in like manner to be considered as a critical process, entirely similar to the first, and it may be designated as a metastasis of the second grade; for the blood is again freed from the still foreign constituent, the pseudo-plasma. Whether this deposition, immediately critical with respect to the blood, which however draws the organ in which the deposition is made into a morbid state, is attended by favorable or unfavorable consequences with respect to the system in general, depends entirely on the place where the deposition takes place. If we suppose, for instance, that the first metastasis took place in the pulmonary tissue, and the second under the cellular tissue of the arm, whereby an abscess was formed, which broke of itself spontaneously, and healed up in a short time, every one will admit that the first metastasis was very unfavourable, the second very favorable: for by the absorption of pus from the lungs these organs were freed from the pathological state into which they would have been inevitably drawn. In the following case, which I myself saw, the very reverse took place. In a scrofulous boy, a tense tumor, about the size of a walnut, had formed beneath the mastoid process, without any exciting cause whatever, save the scrofulous diathesis. The external skin was not at all reddened; very little pain was felt. This tumor, which evidently contained pus, became larger every day, so that at length it attained the size of a man's fist. As the boy's friends would not allow it to be opened, cataplasms were applied, in order to cause the abscess to break. But, instead of this, a very rapid diminution of the tumor followed, in consequence of the pus being absorbed. When this was almost entirely terminated, purulent depositions were observed to take place in the lungs; vomica formed after vomica, and the boy died of scrofulous phthisis. Obviously the first metastasis here was a favorable critical deposition of pseudo-plasma; if the abscess had opened externally of itself, or had been opened by artificial means, the boy would probably have been cured of his disease, instead of dying in consequence of the pus absorbed from the abscess being deposited in an organ which was readily destroyed thereby, the organ also being a vital and very important one. In this case the second metastasis was accordingly an unfavourable critical deposition. The favorable or unfavorable termination of a purulent deposition depends in general on the greater importance of the organ affected; then, whether this be an excretory organ or not, whether the pus can be readily removed from the body, and lastly, whether the

---

mentitious, non-nutritive fluid is coagulable. Puruline (as urine, fæces, &c.) are characterized by an absolute want of coagulability, and as being non-nutritious, must necessarily be excreted." This apodictical and peremptory assertion unfortunately wants decisive proofs; nor has Von WALTHER followed up the enquiry, as to what becomes of the pus in the cases wherein it is not eliminated, as pus by the excretory organs, nor with respect to the ways and changes it must first run through before it can be evacuated by the excretory organs.

organ, in which it was deposited, can be readily drawn by its contagious properties into the same process through which it had itself passed. In these properties, however, may be found the natural causes why pus formed or deposited in an important organ makes a passage for itself externally. (Congestive abscesses.)

Further, the process, which is excited in any tissue whatever by the absorbed pus, in consequence of which it is again deposited, is twofold, according as the pus is absorbed as pus, or only its proximate principles (molecular fibrin.) If the entire pus-corpuscles have been taken up in the way above-mentioned by the venous system, they either reach the heart and pulmonary artery into the capillary structure of the lungs, or in case the purulent deposition was seated in organs, whose veins formed the vena portæ, they reach through these into the capillaries of the liver. Now, as the pus-corpuscles are in general too large to pass through the fine capillary net-work in the lungs and liver, it is evident that for this reason they must begin to stop here, to accumulate and to occasion a stasis, as foreign irritants. These, then, form new pus-depositions, which progressively become greater from the constituents of the blood and of the parts of the tissue capable of it. From this simple cause it is readily explained why secondary abscesses form so frequently in the liver and lungs. If the pus deposited in the liver and newly-formed is taken up by the hepatic veins, these bring it into the heart and into the lungs. If it is taken up from these by the pulmonary veins, it may be deposited in every other organ to which the blood comes from the left ventricle. In this case secondary abscesses may form in the brain, in the kidneys, in the spleen, &c., and pus may even be discovered in the urine. If, on the contrary, the absorbed pus-corpuscles exist in the blood as molecular fibrin, as may most frequently occur, as it is very possible, that the pus-corpuscles themselves, whilst in the blood, may, through the influence of some circumstances, be broken up into separate molecules, the deposition of this molecular fibrin with serum is very easy. As these individual fibrin-corpuscles are still smaller than the blood-vesicles, they may pass with the utmost ease not merely the capillary vessels, but also the so-called serous, plastic vessels, whose existence cannot be at all denied. The pathological process excited by the molecular fibrin, appears to consist merely in a stasis, in consequence of which the serum, together with the fibrin, transudes into the serous cavities, or between the cellular tissue and fibrous membranes. Where this stasis and transudation of the fibrin-containing serum takes place, depends solely on the locus minoris resistentiæ. Otherwise it is not easy to see why, as the molecular fibrin comes in contact in the course of the circulation with all the organs, in one individual the lungs, in another the brain, &c. become the seat of the deposition.

As I myself and some other persons were the first who observed this fibrin-containing serum, it appears to me very probable that the nature of what are called cold or lymphatic abscesses, the so-called cachectic abscesses, the phlegmasia alba dolens, and a series of other pathological processes, consists in this—that amorphous masses of protein-like (fibrin) matters become deposited in the parenchyma of several organs, which matters subsequently run through a cycle of changes, in consequence of which the affected organ is destroyed.—(Morbus Brightii, Tuberculosis, Scrofulosis, Furunculosis, &c.) Hunter (Blood, &c.) understood right well the nature of those abscesses, as he mentions a number of signs or marks which he had obtained by experiment, whereby they are distinguished from true abscesses, and for this reason he calls them abscesses in parts. Such a case, where, in consequence of purulent absorption, a phlegmasia alba dolens was occasioned, I myself once witnessed. It occurred in the case of a soldier, who laboured under pleuritis with considerable plastic effusion into the left side of the chest. At the time of the second venæsection the tendon of the biceps was injured, and whilst those physical symptoms were declining, which denoted the fluid exsudation, there soon arose an enormous swelling of the entire left upper extremity as far as the mamma, during which the external skin became of a bright red colour, and doughy. By suitable treatment the tumor gradually diminished, without pus having formed, and when the patient died, about the 14th day of the disease, the arm had the same strength as the other. On the contrary, the entire left lung was so compressed, that it was not an inch in thickness, and besides, it was covered here and there with purulent deposits. Adhesions existed only on the posterior part of the lung. I conceive this to have been a case where the exuded pseudo-plasma, which probably had become changed in a great measure into pus, was re-absorbed, and deposited from the blood into the vessels and between the sheaths of the muscles and into the cellular tissue of the arm, in consequence of the latter having become a locus minoris resistentiæ by reason of the injury done to the vein and the fibrous structure; a circumstance for which I was unable to account to myself at the time.

On the quantity and quality of the pus, as also on the irritability of the individual and some other circumstances, it depends what influence and irritation its constituents shall exercise on the nervous system. If neither the quantity nor quality be very abnormal, there may be no constitutional disturbance whatever. If, on the contrary, the quantity be very considerable and the quality be very much deteriorated, as in the abscesses of cachectic persons, in typhus abdominalis, puerperal fever, the last stage of phthisis, &c. then such an impression manifests itself on the blood and the nervous system, that adynamic or putrid fever, delirium, subsultus, &c. are soon developed. The most favorable way in which the absorbed pus-corpuscles or the molecular fibrin can be removed from the body, is through the excretory organs, the skin, intestinal canal, and kidneys. With respect to the latter, the cases are very numerous where real pus-corpuscles are observed in the urine: in variola at the time of exsiccation, in empyema, pleuritis, pneumonia, &c. at the time of resolution, purulent urine has been observed. It is evident that in these cases laceration must have taken place in the renal vessels, in consequence of which the pus-corpuscles were pushed forward into the calices of the kidneys—that the absorbed molecular fibrin existing in the blood at the time of the pus-absorption was in a similar manner eliminated by the kidneys I have already stated. As the molecular fibrin may pass very well through the finest capillary vessels of the kidneys, as well as of the renal canals, its passing into these takes place very simply by transudation, which is further assisted by the affinity which the molecular fibrin, existing in the blood as excrementitious substance, seems to have for the renal cells of the renal canals, as well as by the appropriating power of the latter. I cannot help again thinking of the case mentioned by H. Nasse in his Physiological and Pathological Enquiries, in the person of an ecclesiastic whose urine for a long time contained so much fluid fibrine, and frequently fibrin coagulated in the bladder, that the urine after perfect coagulation looked like jelly. And with all this the patient became very little or not at all emaciated. Neither myself nor Nasse were able to find blood-corpuscles in this fibrinous urine, a proof that no laceration took place of the capillary vessels in the kidneys.

Thus either the pus-corpuscles or the molecular-fibrin are directly eliminated through the kidneys, and good proofs of this may be adduced, or these protein organic matters, by coming in contact with the oxygen of the air in the lungs and the capillary vessels of the external integuments, undergo such a chemical change, that they are broken up either immediately in the blood, or by the changing power of the excretory glands (kidneys, cutaneous glands) into the products appertaining to the regressive transformations, as urea, urate of ammonia, lactate of ammonia, uric acid, phosphates and sulphates, or into crystallized triple phosphates by combination with ammonia which has been set free. This, and the occurrence of coagulable urine, are cases where practitioners were unable to account to themselves, as to what became of the pus from abscesses, which were so large that perfect absorption was not to be expected; but this could only be concealed for this reason, because they neglected to examine the urine of their patients.

I am now come to the point where I stopped in my paper on the urinary crisis in inflammations, published some time since, namely, that of proving that the pathological positive changes observed for the entire duration of a disease, stand with the transformations of the blood-constituents recognised as the materia peccans in the relation of effect to cause. This cannot be shown better than in the case of patients whose disease is not accompanied by fever, because one might feel himself justified in setting down to the account of such reaction the anomalous constituents in the urine. The urine containing fibrine, urate of ammonia or crystals of uric acid, must be indebted for these more anomalous constituents only to the absorbed pus; for in the patients, whose history I shall briefly notice, there are no circumstances which could have produced them in any other manner. Sediments of urate of ammonia or of pure crystals of uric acid can of themselves be but seldom produced in the urine of healthy men, and they are then indebted for their production to a combination of circumstances, which leave no doubt, that on the part of the vital process which comprises the regressive transformations, changes have gone on, which are still within the limits of health.

The series of changes which the fibrin undergoes through the action of the oxygen in the blood, and probably also in the cells of the excreting glands, are, according to Liebig, probably as follows: the fibrine is broken up first into urate of ammonia, choleic acid, sulphur, phosphorus, and phosphate of lime; the urate of ammonia by the further action of oxygen into urea and carbonic acid; the choleic acid into carbonic acid and carbonate of ammonia; the sulphur and phosphorus into sulphuric and phosphoric acids, which, combining with an alkali or earth, form sulphates and phosphates. If it should happen that the quantity of oxygen taken in is not sufficient completely to accomplish this cycle of changes, then, instead of urea, either urate of ammonia, and if the ammonia have entered into any other combinations, pure crystals of uric acid, or fibrin itself, appear in the urine. By taking into account those circumstances which made the oxygen taken in insufficient, we shall always be able, in the particular case, to account to ourselves with respect to the origin and appearance of those abnormal constituents of the urine: these circumstances may for instance be founded on the insufficient quantity of oxygen contained in the air, in a deficient quantity of oxygen taken in, the causes of which may lie in a morbid state of the chest, of the lungs, of the respiratory nerves, in a defect of blood-vesicles, &c. or in a morbid state of the skin, or in a depressed state of the peripheric circulation, or, in fine, in too great an accumulation of excrementitious substances. Whether the pus-corpuscles are in like manner capable of running through these changes, I shall not venture to determine; but that the molecular fibrin does so, of that I have satisfied myself by a series of experiments.

In some cases of disease which I shall mention presently, I shall take the opportunity to adduce proofs in support of my assertions; though such cases might inspire no interest, if they had been observed, as unfortunately such cases have been constantly observed, they however, possess this advantage, that of allowing us to look into the nature of the pseudo-plastic processes in general.

The first patient on whom I observed that at the time of the ripening of the abscess the urine evinced morbid changes, was a soldier who was after being affected with erysipelas of the face. At the period of the decline of the disease the urine, which previously yielded a lateritious sediment, had now become quite pale and watery, without being coagulable, and the desquamation of the skin extended from the head over the entire body. At this time, the circulation in the peripheric vessels of the skin was almost entirely depressed; the skin was dry, friable, parchment-like and never transpiring. As the patient had several abscesses at the time, on the head, on the neck, on the thigh and leg; every time this or that abscess was brought to maturity by the application of cataplasms, I found the urine coagulable through its fibrinous contents; now, if the abscess was opened, it no longer contained any fibrin, but, in one or two days, uric acid crystals were deposited at the edge of the urine-glass. These abscesses may be considered as supplementary crises of the erysipelas, though their origin may be derived from another source: none, however, will deny that the pathological change in the urine corresponds with the absorption of pus. The circumstances here, which rendered the action of the oxygen on the blood insufficient, are in the first place, the quality of the skin, the vessels of which carried very little blood; the respiratory function, which was much depressed through the debility of the patient, and the slowness of the circulation, and, lastly, the excessive mass of the fibrin in the blood, which was to be acted on. For we see that, after opening the abscesses, whereby the further absorption of pus was put a stop to, no longer fibrin, but uric acid, appeared in excessive quantity in the urine, whereupon the urine again became normal. Güterbock mentions, from Schönlein's Clinique, a case of rheumatism of the abdominal muscles, where an abscess formed, but the pus had evacuated itself externally through the rectum. Here no sediments were observed in the urine, for this simple reason, that the pus was not absorbed; but whether, before the discharge of the pus, the urine contained anomalous constituents, has not been stated. It might be possible, however, that by the application of cold, sediments of urate of ammonia, or of uric acid crystals might be occasioned in the urine. The patient, of whom I have made mention, had at the time no fever, nay, rather a retardation of the pulse, and his skin was not at all in a state of transpiration. His blood, as well in consequence of the treatment (a blood-letting and antiphlogistic medication,) as also by reason of the entire morbid process, (phthisis,) contained but very few blood-vesicles. In this lay another cause why the absorption of oxygen took place so insufficiently.

II. The soldier, Graaf, with brown tint and black hair, had in the March of this year a porriginous eruption of the back, and at the same time periostitis of the portion of the occipital bone, which passed quickly into suppuration. Three other soldiers were suffering at this time under the same form of disease. The patient came, on the 16th of March, into the Lazareth; he had no fever. To the tumor, which was now of a considerable size, and tolerably hard, cataplasms were applied; the patient took no medicine internally. From the 17th the urine was of a yellow turbid appearance, was very acid, and threw down a very copious precipitate of crystallised uric acid. Notwithstanding this, it coagulated on boiling, and contained fibrin. Three days after, it became alkaline, and besides the uric acid crystals, it contained also triplephosphate. On the 18th, the swelling had become considerably smaller and less painful. The urine was very much saturated, and, at 2° R., threw down a lateritious sediment, which the urine of the other soldiers, subjected to the same regimen, did not do. Fever had not even yet become established. On the 19th, the tumor had almost entirely disappeared under the use of cataplasms; the urine threw down no sediment, but on boiling it became very turbid, and contained fibrin. Three days after it was alkaline. On the 20th, 21st, and 22d, the tumor was still further diminished; nothing anomalous in the urine. On the 24th, in consequence of taking cold, the inflammation was again become violent, and the tumor was as large as at first. No fever. The urine looked somewhat turbid, but did not coagulate on boiling, but rather became clear thereby; on cooling, it threw down a lateritious sediment. The cataplasms were continued. On the 25th, the tumor had become larger; the urine was of an amber colour, very acid, threw down a sediment on becoming cold, and did not coagulate on being heated.

On the following day, the tumor began to fluctuate, and had become tolerably soft; at 5° R. the urine threw down a lateritious sediment. On the 27th, the urine had a sediment, and did not coagulate; a cloud floated on the surface, which consisted of mucus-corpuscles. The urine was very acid; but in four days it became alkaline. On the day after, the abscess was opened, and good pus discharged: nothing abnormal in the urine, and the abscess healed very rapidly under the use of cataplasms.

We here see that, about the time when the tumor became smaller, and when, therefore, the absorption of pus was going on most rapidly, (on the 18th and 19th,) the urine contained the most anomalous constituents, fibrin and urate of ammonia; after the absorption had terminated, we see the urine quite normal; (from the 20th to the 23rd). Subsequently, on the relapse, at the time when the abscess was again approaching being ripe, the urine again threw down the lateritious sediment.

It would be very interesting, if we could ascertain, by observation, how long a time an abscess requires, before the pus contained in it becomes perfectly absorbed, and eliminated by the excretory organs; and whether this whole cycle of transformations would be confined with the three and a half days' type, as we see in other acute diseases. But such a natural method of cure would be a great reproach to art, as by opening and discharging the abscess we possess the means of completely putting a stop to the morbid process excited by the pseudoplasma, whereby its deposition amongst the external integuments and the superficial glands would be occasioned. For either a second deposition may follow the absorption of the pus, and that, too, into an important organ, or induration takes place. This latter termination seems to me to arise from the circumstance of the fluid parts being first absorbed from the abscess; then the more organized matter is taken up, so that at length the phosphoric salts and the phosphate of lime with some organic matters remain behind. This is the origin of most concretions. The same process takes place in what Bayle calls the phthisis calculosa. For the chalky concretions which contain 96 per cent. of saline and earthy matter, and only four of animal matter, are in all cases formed by the absorption of the fluid tuberculous mass.

These considerations respecting the absorption of pus, have been communicated thus fully for this reason, that they may serve as a sort of commentary for a series of other morbid processes, wherein the same occurrences take place in a similar manner, as, for instance, in pneumonia, pleuritis, erysipelas, &c. but in which, by reason of their more complex nosology, it cannot be demonstrated so clearly that the pathological changes in the urine are closely connected with the organico-chemical transformations of the materia peccans.

---

### REMARKS ON LIEBIG'S "ORGANIC CHEMISTRY IN ITS APPLICATION TO PHYSIOLOGY AND PATHOLOGY;" as also on the Application of Chemistry to Medicine in general. By *Professor Schultz*, of Berlin.

ORGANIC Life is every where accompanied by chemical phenomena, and Medicine, in consequence of this circumstance, is every where coming in constant contact with Chemistry. But it is of great importance correctly to understand the nature of this contact, in order to make a judicious application of Chemistry to Medicine. Life being accompanied by chemical phenomena, many persons imagined, and still imagine, that Life itself is chemical—to this class Liebig belongs. What is called "the application of Chemistry to Physiology and Pathology" is really the substitution of Chemistry for Pathology and Physiology. This is the true sense of all modern treatises on Chemical Physiology, Physiological Chemistry, &c. &c. They contain chemical explanations of Life itself, and such an attempt at chemical explanations of Life in health and in disease is

Liebig's work. Instances of a somewhat similar tendency have been witnessed to no end in medicine; at all times a desire to find the vital philosopher's stone in chemistry has been evinced, and various explanations of Life have been given —modified only by the existing state of chemistry. Liebig's Theory of Life is essentially a chemical combustion-theory, wherein Life is compared to fire and analogous chemical phenomena of oxidation. Liebig has divided his work into three parts: 1. The chemical process of respiration and nutrition. 2. The chemical process of the transformation of tissues. 3. The phenomena of motion in the animal organism.

In the first part, Liebig says, that the greatest physiologists have been far removed from a knowledge of the laws of purely animal life. "None of them had a clear idea of the processes of development and nutrition, nor of the true causes of death. They accounted for the most recondite psychical phenomena, and were not able to tell what is Fever, and how bark acts in curing it. The solution of these points is reserved for Chemistry." Liebig's mode of explaining these matters, is as follows. Every motion, every manifestation of power, is the consequence of the transformation of the tissues, or of their substance; every idea, every feeling is followed by changes in the chemical quality of the secreted juices; every thought, every sensation is accompanied by a change in the chemical composition of the cerebral substance. From this it must be inferred, that the chemical changes are the cause of the vital powers. "The only known and ultimate cause of vital action in the animal, as in the plant, is a chemical process." (See Lieb. Eng. Trans. p. 6.)

The food serves the purpose of compensating for the consumed material in the body, and thereby of producing power. The power arises from the material being set in motion from its static equilibrium, and again passing into static equilibrium. Chemical Affinity is the power that sets in motion. Its action is more particularly seen in the phenomena of Respiration and Nutrition, and all the vital properties arise accordingly from the mutual action of the oxygen of the air, and the constituents of the food.—(See L. p. 10.) Life consists in a constant waste of substances, and conformably with this in a constant supply of substances in the form of nourishment, and of the oxygen of the atmospheric air, which serves for the combustion of the materials of nutrition. It is readily seen that this view is a repetition of the old Aristotelic and Baconian consumption-theory. Bacon, in his celebrated work, "Historia Vitæ et Mortis," which served as the groundwork of Hufeland's Makrobiotik, brought forward the same theory, and the reparation of the body by means of the food. The theory of combustion adopted here is modified only through the progress of chemistry. Liebig naturally enough takes it up in the spirit of Lavoisier's discoveries, about which Bacon knew nothing.

Liebig's book next treats of the subject of statics, wherein the pound and the cubic foot of oxygen are calculated according to Lavoisier's well-known experiment, which are the quantities the lung takes in during respiration, in comparison with the quantities of Hydrogen and Carbon, which are present in the expired and vaporized water, and in the expired carbonic acid. The supply of carbon and hydrogen is effected only by nutrition. An adult consumes in his food daily about 13 ounces of carbon, and in order to burn this and convert it into carbonic acid gas, about 37 ounces of oxygen. The nitrogenous constituents of the food, as albumen, caseine, come forth again after this transformation, as uric acid and urea in the urine; but Liebig has instituted no comparative calculations between the quantities of nitrogen in the urine and in the consumed food; had he done so, he would have found, that the urine contains only an inconsiderable quantity, compared with the nitrogen contained in the food. His calculations have reference only to the combustion of carbon. Liebig views the constituents of the blood only as fibrin and albumen, and considers that these are formed solely from articles of food containing nitrogen. The substances containing carbon, as butter, sugar, starch, can, according to him, supply none of the constituents of the blood (p. 52). They merely serve the purpose of supporting the combustion. But with respect to the form in which the carbon is communicated to the lungs for combustion, we are told nothing.

It is evident here, that Liebig seems to forget important physiological facts, viz. that not merely fibrin and albumen, but fat and colouring matters are chemical constituents of the blood, and that the fat is contained in the young vesicles in excessive quantity, which are formed from the fatty globules of the lymph.

By means of his combustion-theory, Liebig now endeavours to account for two phenomena, namely, the production of warmth, and the formation of fat in animals. Here Liebig strives to make Lavoisier's theory, according to which animal heat is occasioned by the combustion of hydro-carbon in the lungs, directly available against those, who would ascribe the production of warmth to the nervous system. However, to contradict the opinion of the origin of heat being from the nervous system, and to prove that heat has its origin from the combustion of oxygen and hydrogen, are two very different things. Liebig's argument for the latter view of the matter is as follows: the animal body is like an oven, which we supply with fuel. If it is cold, we must, in order to keep the oven warm, introduce much fuel: if it is warm, the oven does not cool so much, and we require less fuel. Accordingly, in Winter, and in the Polar Regions, the necessity for food containing carbon and hydrogen increases. In warm climates the necessity for food is not at all so great, because the body does not require to generate so much heat. The warmer we clothe ourselves in Winter, the less food we require, because so much heat is not lost. If we went naked, like the Indians, we should require to take much more food as fuel.

The Neapolitan cannot take in more carbon and hydrogen in his food than he exhales, and the inhabitant of the Polar Regions cannot inhale more carbon and hydrogen than he has taken in his food. The predacious animals of the Northern climates surpass all others in voraciousness. These, and other inferences, Liebig proves, from the well-known phenomena, that man lives more moderately, in general, in warm than in cold countries, but rather unsatisfactorily: partly because they are brought forward with considerable contradictions, and partly because they are laid down without a knowledge of their connexion. The contradictions consist in this, that Liebig, according to his theory, at once admits that it is only vegetable food, food abounding in carbon, that is principally adapted for combustion, animal substances, containing nitrogen, being less suited for the production of heat; and still he brings forward the voraciousness of the predacious animals of the North for flesh, and, consequently, for nitrogenous food, which, according to his own theory, cannot burn, as a proof, that here a powerful combustion-process must take place in order to generate heat.. According to Liebig's theory, in cold countries no flesh at all should be eaten, but only vegetables, because these latter favour the combustion-process powerfully, whilst flesh favours it but very inconsiderably.

But the phenomena are stated without a knowledge of the connexion, because the general observation, that the inhabitants of cold countries eat much, and those of warm countries eat little, is by no means correct. The truth is, that in cold countries more flesh and less vegetables, in warm countries, on the contrary, more vegetables and less flesh is eaten. There surely is no great moderation in a man in Persia or Arabia eating every day from 20 to 30 pounds of melons and other fruits, and the large-paunched Negro does not display this assumed abstemiousness of the inhabitants of the Tropical climates. Further, this combustion-theory contradicts, in the most decided manner, ordinary experience. According to Liebig's theory, vegetable food should be heating, and animal food cooling. But every day's experience, both in the case of men, and animals, teaches the direct contrary. Meat diet has a heating, vegetable diet has a cooling effect. Accordingly, the roots, fruits, and seeds which, according to Liebig's views, promote combustion, diminish the heat of the body; whilst the slightly combustible muscular flesh increases this to a high degree. Graminivorous animals possess but a small degree of bodily heat, and for this very reason, they cannot hold out in high latitudes; the carnivorous animals, of all countries, are from two to three degrees warmer than the graminivorous. It is the same with man. The inhabitants of the Polar Regions sustain themselves merely by the heating effects of flesh diet; they eat scarcely any vegetables, and would become frozen, if they did. In the Tropical Regions, the inhabitants keep themselves cool by living on vegetables, and avoiding, or cutting short, the proportion of flesh diet.

Liebig has, to be sure, stated that, in consequence of the small amount of carbon contained in flesh, men and animals were obliged to consume large quantities of it. Now this is also experimentally incorrect: we rather see all carnivorous animals take much less food, for which reason, too, they are very slender; whereas, on the contrary, the mass of food of herbivorous animals is enormous; their digestive organs are also constructed so as to suit these great masses of food. Liebig should have calculated what heat an elephant must have, according to his theory, who consumes daily 100 weight of hay, potatoes, and corn. But still, elephants are so very much affected by cold among us, that they can scarcely hold out without brandy.

These, however, are not the slightest objections against the production of heat by combustion through the medium of the Respiration. The heat in certain parts of the body is raised or depressed independently of the respiration. Paralysed limbs become cold, whilst the respiration still continues. In inflamed parts of the body, the heat rises from four to five degrees: the stomach becomes two degrees warmer in digestion: when the depuratory substances are retained in the blood, for instance, after removal of the kidneys, the heat rises six or eight degrees; the same thing occurs in several cases of fever, where the respiration is not at all raised. How can combustion take place here?

The theory of the combustion of hydrogen militates against several physiological experiments. Watery vapour is exhaled from the lungs when no oxygen gas whatever was inspired; when hydrogen or pure nitrogen was inhaled. How could it be formed here by combustion? In another work of mine, I have demonstrated that the source of heat lay in something altogether different, namely, in increased excitation of the blood-vesicles, and in the formative process increased thereby; hence, animal food abounding in nitrogen, which promotes the formation of the blood-vesicles, likewise increases the heat without reference to combustion; whereas, on the contrary, vegetable food abounding in carbon, because it rather promotes the formation of the nuclei in the blood-vesicles, and diminishes the vesicle-formation itself, does not favour the production of heat.

Liebig's theories regarding the formation of fat, and the consumption of fat, are disposed of similarly. According to these theories, the fat is to be the fuel for the organic heating process. According to Liebig, the hungry man wastes away, because, for want of fuel in the food, the oxygen burns down the fat. Now, even though one should ascertain the way by which the oxygen is to reach the fat, there still remains this difficulty, viz. that it is not the fat alone by whose disappearance the hungry man becomes emaciated. All the tissues, even those containing but little carbon, as the muscular, nervous, and albuminous tissues, which cannot at all burn, become absorbed, and not the fat merely. The same action which absorbs the incombustible nitrogenous tissues, absorbs the fat also. Liebig says, that for want of oxygen, and from diminished respiration, as in animals in a state of rest, fat is formed because all the carbon cannot then be burned down. This is not true. It is quite an error to suppose, that animals fattened in a state of rest, breathe little and form but little heat; on the other hand, the respiration and the formation of carbonic acid is very considerable, the heat of the animals is very great, and, for this reason, they fatten better in cool weather, than in great heat. The formation of fat depends rather on the action of the digestive apparatus, the quantity of the bile, and the way in which the chyme is neutralized by it. The state of rest in which animals may be kept, acts much more on the digestive process, than on the respiration. And besides fat is something else than mere carbon. In the second part of his work entitled "Transformation of the Tissues," Liebig treats of the various chemical transformations of Protein, a substance which he endeavours to shew to be the fundamental element of all organic structures; the latter being merely modifications of protein. He compares the chemical composition of protein with that of the other organic structures, and makes it appear that they all agree in consisting of certain proportions of carbon, oxygen, hydrogen, and nitrogen. This, however, is mere chemistry, and has nothing whatever to do with physiology. The real differences of the organic structures lie not in the chemical material, but in the organic form, in the globular, fibrous, tubular, &c. formation.

In the third part, entitled the Phenomena of Motion in the Animal Organism, Liebig endeavours to find the fundamental seat of the moving power in the chemical change of substance. As the galvanic power, which is formed by the contact of two metallic plates with an acid, is dependent on the continuance of the chemical action, so the vital power, according to Liebig, is connected with the change of substance, which arises more especially from the chemical action of oxygen. Thus, life is to consist in a waste and new supply of substances through changes of substances. Now, as different individuals waste in twenty-four hours an unequal quantity of the parts of their bodies, a state must take place, where the voluntary motions are suspended, in which no waste takes place. This state is sleep. On waking again, the equilibrium of wasting and renewal of substances is restored. This is the chemistry of sleep. We have a similar chemistry for syncope, paralysis, &c. Heat accelerates the change of substance in the living body; it occasions, therefore, an increase of the mass, and of the vital power in the different periods of life; cold, on the contrary, suspends the change of substances. This furnishes us with the chemistry of growth, and of the decline of the vital power in old age.

Besides all this, Liebig has given us a chemistry of disease. According to this, disease is a disturbance in the equilibrium between supply and waste of substance. As in reference to the normal supply and waste of substance, Liebig compares man to an oven to be heated, so, in the case of disease, he uses the comparison of a self-regulating steam-engine. If an intense heat occasion an abnormal change of substance in the case of cerebral congestion, cold water is employed, in order to reduce the heat, and moderate the change of structure. When, in consequence of a morbid change of structure, a greater proportion of power is generated than is required for the production of normal motion, acceleration of the involuntary motions take place. This is fever. When the excess of power, occasioned by the change of substance, is thrown upon the voluntary motion, a febrile paroxysm is occasioned. In all these cases, we have only to see, that fever does not consist merely in accelerated involuntary motion; further, that it is not all the motions, but only those of the heart, that are accelerated, and not those of the intestinal canal, and that the paroxysm is a somewhat different thing from accelerated voluntary motion.

In conclusion, we have also a chemical theory of respiration. This is founded on the iron contained in the blood, and on the reciprocal action thereby occasioned between the blood and the oxygen of the air. According to Liebig the blood-corpuscles of the arterial blood contain a combination of iron saturated with oxygen; this, while passing through the capillary vessels, gives a portion of its oxygen to certain constituents of the animal body, in order to produce the change of substance and the secretions; but the greater part of its oxygen is combined with the substances of the body, that have now become lifeless, which then make their escape out of the body as oxides. On the way from the capillaries to the heart, the iron combinations of the blood-corpuscles, which gave away the oxygen, take up carbonic acid gas; the blood thereby becomes venous and so reaches the lungs. Here the organic iron-combination receives a compensation for its lost oxygen, and in consequence of the new accession of oxygen in the lungs, the carbonic acid of the blood is given off. All the matters present in the venous blood, which have an affinity for oxygen, except the iron, become more highly oxidized; a quantity of carbonic acid is produced, of which there is always a portion present in the blood. This carbonic acid is partly combined with soda, and is equal in both kinds of blood, because they have the same temperature. (Now they have not the same temperature, the Arterial being one or two degrees warmer.) But more carbonic acid can be produced in the arterial blood, because it takes in oxygen. According to this, Liebig establishes two processes of oxidation: the one being in the lungs, whereby the temperature of these organs is kept up, and the other in the periphery of the body, whereby its temperature is sustained.

Now the principal thing to remark here is, that physiological observations prove, that the absorption of oxygen through the blood-vesicles proceeds not at all from the chemical matters therein contained (which yet could only be the colouring matter), but it is occasioned solely by the vital contractility of the vesicular membranes, which include the colouring matter; further, that physiological observations show, how the formation of the colouring matter in the vesicles is only an effect of the absorption of oxygen, inasmuch as the originally colourless vesicles, which certainly do not contain iron, become red only through the absorption of oxygen. (This we have proved in our Essay on the Circulation.) Both in the embryo and in the formation of blood during or after digestion the vesicles are at first quite colourless, and they only become red in proportion as they absorb oxygen. We have further shown that the capability to absorb oxygen diminishes in the older vesicles, according as the colouring matter and the contained iron increases; accordingly those vesicles, which, according to Liebig's theory, should possess the greatest power of absorbing oxygen, because they contain most iron, have the least, and ultimately no power at all, of attracting oxygen.

In all these hypotheses it is not taken into consideration, that the human body must become dissolved like a saline solution, if it merely consist of substances without organic form, organic form being presupposed as necessary to all vital action. It is further to be considered that all substances brought to the body become assimilated to the organic form, and that the quality of the substance must be annihilated, before the body can receive nutrition from it. It is only from the organic form the vital powers can be derived. This it is which can re-act against external stimuli; but in such case it only shews a change of form, and no change of substance, because the quality of the substance is entirely suspended. It is not till after the cessation of life and the destruction of the organic form, that the qualities of the substance are again brought forward.

The absorption of oxygen in the lungs, and the reception of nourishment, do not then produce life, as Liebig assumes, through the transformation of substances, but the self-excitation of the organic form occasions their reception and elaboration. In so far as a change of substance takes place at the commencement and at the termination of life, at entering and making an exit from her portals, this is the effect and consequence, and not the cause of life. In the living organism itself there is only a change of forms. It is only the vital conditions and the vital residues which indicate chemical actions. Nor must we close our eyes to the important fact, that, in undertaking a chemical experiment with an organized structure, the organic form disappears in the substance, and no vital excitation any longer remains in it; whilst, on the other hand, it is impossible to call forth any vital action from the chemical substance.

Because chemical phenomena go hand-in-hand with life, some persons have been erroneously led to believe, that these phenomena exist in life itself. But such is not the case. There is an eternal struggle between life and death, which the body has to sustain with the external world, whilst it assimilates the various substances to itself. The essence of life does not lie in these substances, nor in their ingress or egress from the organism.

If we now consider more attentively the contents of Liebig's book, we find that it treats of the mechanism of the actions of organic life, as also of their chemistry. Liebig, in taking the mechanism of the vital actions (the manifestations of power), and the chemistry of the vital conditions and of the vital residues for life itself and its causes, is involved in a delusion, by which, in the successive relations of life to the external world, he is led to take that which is consecutive and chemical for that which is originally vital, and vice versâ, just as he everywhere confounds causes and effects with each other, and constantly takes a part for the whole, by identifying individual chemical and mechanical relations to the organism with the nature and essence of the entire organism, whilst he entirely overlooks the wide field of the truly organic and vital phenomena. Liebig is constantly under a gross delusion, fancying that he is engaged with physiology, whilst he is really stuck fast in the very midst of chemistry. In consequence of the erroneous idea, that the vital phenomena must be explicable from the chemical theory of change of substance, he everywhere converts the means into the end of organisation; the end of organic life being altogether overlooked and unnoticed by him. All he says, regards only the ways and means whereby the organization attains its end.

There is not a doubt that the knowledge of the ways and means which organic life employs to attain its end, appertains to the knowledge of life. Care should be taken duly to distinguish means and end, and not to confound them, as Liebig has invariably done. Supposing that what Liebig asserts in the first part of his book were correct, viz. that the supply of air and food were in a sort of static equilibrium with respect to each other, so as to become mutually dependent on each other, still it does not follow, as Liebig will have it, that the true ends of life consisted in the interchange of substance between the oxygen of the air, and the carbon and hydrogen of the food, and that the living body is nothing else than an oven or a steam-engine, to which Liebig so often compares it. The living end might make use of such mechanical and chemical means, without being mechanical and chemical itself.

The imperfection of Liebig's book goes much farther on this point, than might appear at first sight. For this dependence of the inspired air on the food, and the mutual demand for the carbon and hydrogen of the food and the oxygen of the air do not exist in nature, in the manner that Liebig states. We shall mention one instance. Liebig says, the time in which a starving man dies, is regulated by the state of the fat of his body, by the amount of his motion, by the temperature of the air, and lastly, it depends on the presence or absence of water. This is explained, as if the starving is a combustion-process, wherein death follows; the fire goes out, because no more fuel, *i. e.* fat, which for want of food serves as fuel, exists in the body. On the other hand, the fat in the body is consumed sooner when the respiration is excited by motion and exertion, and accordingly the fire goes out so much the sooner, the greater the draught of air is. The more deficient the water is, the more is the combustion accelerated. The peculiar cause of death by starvation is therefore the respiratory process. This is Liebig's mode of expressing it.

Unfortunately, for all this reasoning, the first and leading proposition is incorrect; scil. that the time in which a starving man dies is regulated by the quantity of fat collected in his body. For graminivorous animals, in which alone much fat is formed, no matter how fat they may be, always die in a much shorter time than carnivorous animals, in which, generally speaking, scarcely any fat is formed. A meagre, carnivorous animal, a dog, or a cat, may fast from four to six weeks, before it dies; most animals of prey are forced to remain hungry for the length of a week, before they can alight on any prey; and during this long time they sustain themselves without any fat. A graminivorous animal, a fat sheep or a fowl, with all their fat, cannot fast much beyond eight days. This is the real physiological fact, one however which suits very badly the chemical starvation-theory. That during starving fat is absorbed, if fat exists, is quite true, but it is not merely the fat, but all the other organic structures are also absorbed, the albumen, the muscles, the nitrogenous substances in general, which are not at all combustible.

But supposing that during hunger fat only were absorbed, and that the oxygen really consumed the fat, could death be occasioned by the combustion of fat? Would the peculiar cause of death, as Liebig says, be the respiration? According to his own theory this would be impossible. Liebig's leading proposition is, that organic life is founded in the change of substance, and the change of substance in the combustion-process through the respiration. In other words, then, the respiration is to be the real cause of life; to prove this proposition, is the earnest endeavour of the entire of Liebig's book. But he now tells us that in death by hunger the respiration is the cause of death: so that he makes no distinction between life and death.

Now what is the ultimate end of starvation? It is this; that the organic excitement is destroyed, and the organic form is lost. But in the case of starved persons this takes place rather through putrefaction than through combustion. Through hunger the entire body passes ultimately into a state of internal decomposition; the lungs exhale ammonia instead of carbonic acid; so much so that the respiration dies, the blood putrefies. Liebig seems not to be aware of this.

Liebig further says, carnivorous animals and man would have too little carbon and hydrogen in their nitrogenous food to supply the lung with materials for combustion. They are therefore obliged to consume a great quantity of food merely to create carbon for the lungs. An Indian, when deficient in vegetable food to mix with his flesh-meat, must consume five times as much flesh as vegetables, in order to create the necessary quantity of carbon for respiration. For the same reason it is that lions and tigers are so voracious. In warm countries the respiration is diminished, and therefore man requires to eat less. But here Liebig has allowed himself to be led astray by the ordinary mode of speaking with respect to the voraciousness of predacious animals. Notwithstanding their voraciousness these animals do not eat half, nay, not even the fourth part as much as herbivorous animals, which are eating the whole day, whilst carnivorous animals eat but seldom. This truth is therefore not suitable to Liebig's theory. Hence we may see that the quantity and quality of the food of man and animals must have quite a different end from that of being subservient to the combustion-process. Flesh-meat is difficult to be procured in warm countries; when taken in large quantity, it readily passes into chemical decomposition in the intestinal canal. In tropical climates much vegetable food is required, notwithstanding the diminished combustion (according to the chemical theory.) Liebig with his chemical physiology never comes to the true ends of life. According to his theory, these ends are eating, drinking, digesting, and sleeping. With this physiology he never gets out of the intestinal canal; but constantly sticks there in a state of chemical decomposition. Physiology is by him converted into chemistry, and organic life is accounted for on inorganic principles. It is plain that, however correct the doctrine may be of the chemical changes of various substances outside the body, the end of life must be something else besides that of changing substances, and that no change of substances is ever able to produce the interesting series of vital phenomena, which present themselves in the organism. It is no doubt highly interesting to become acquainted with the relations of the external world and the organic, to see what the organism receives from the external world, and what it gives back to it in return. These relations, however, appertain not to the interior machinery of the organization itself; these relations are not life, but the means and accidents of life.

In conclusion, we must observe that it is not Liebig alone who has become involved in the errors now pointed out. Many others are in the same case. Only Liebig, being less aware of the difficulties which met him in the domain of physiology, or of the dangers to which he was exposed therein, was led away so as to penetrate farther in this erroneous direction than he would otherwise have ventured. Liebig's deserts in the department of chemistry, as such, we acknowledge with pleasure; they possess the highest interest for the physician on the important subject of the residues of life; but we consider it our duty strenuously to oppose the erroneous application of chemistry to physiology, wherein the doctrine of change of substance and of the chemical process is mistaken for the doctrine of the vital process, fearing lest such erroneous application may occasion an injurious re-action on practical life.—*Condensed from the Beiträge zur Physiologischen und Pathologischen Chemie und Mikroscopie, &c. Berlin*, 1844. *By Dr. Fr. Simon.*

---

MENTAL HYGIENE: OR AN EXAMINATION OF THE INTELLECT AND PASSIONS, DESIGNED TO ILLUSTRATE THEIR INFLUENCE ON HEALTH AND THE DURATION OF LIFE. By *W. Sweetser*, M.D. (Reprinted from the American Edition.) Edinburgh, 1844.

THE close and intimate connexion between mind and body, the reciprocal influence so constantly maintained between both, may be said to be self-evident. So obvious indeed is this mutuality, and so palpable is the constant interchange of influence subsisting between our mental and corporeal natures, that they can hardly have escaped the most inattentive observation. We every day see that the functions of either being disturbed, more or less derangement will invariably be reflected to those of the other. There is no bodily frame so strong as to escape suffering, when the mind has been agitated and afflicted; nor any mind so firm, as to remain unharmed amid the infirmities and sufferings of the body.

The object of the work now under consideration is to elucidate the influence of intellect and passion on the health and well-being of the human frame. There is good reason to believe that this influence has been but very imperfectly understood and appreciated in its character and importance by mankind in general. Few have formed any adequate estimate of the sum of bodily ills which have their source in the mind. Even the members of the medical profession, habitually concentrating their attention upon physical, are too prone to neglect the mental, causes of disease; and thus may patients be subjected to the harshest medicines of the Pharmacopœia, the true origin of whose malady is some inward and rooted sorrow, which a moral balm alone can reach. The present work is divided into two parts. Under the first, the author considers the intellectual operations in respect to their influence on the general functions of the body. But the effects which they induce in the animal economy being less strongly marked, and less hazardous to its welfare, than those belonging to the passions, comparatively little space is devoted to this division. Indeed, when pure, or as much so as is consistent with their nature, the intellectual operations can by no means be viewed as an ordinary occasion of disease, or as tending directly to abbreviate the term of human existence. On the contrary, ordinary experience warrants us in believing that a judicious exercise of the intellectual faculties is conducive both to health and happiness. We know there have not been wanting persons, aye, and those persons eminent for learning too, who maintain that the savage is our only natural and happy condition. Thus, we read of man in the golden age of his early creation, dwelling in a mild and balmy climate abounding in vegetable productions suitable to his wants, living solitary, naked, savage; roaming without care or thought the vast forests which he held in common with the brute—we read of him as being then pure, gentle, and innocent, exempt from all those multiform and painful maladies which now afflict and shorten his career. In this state, free from all those lights and shadows of the soul which spring from cultivated intellect, like the brutes, he was happy in the bare consciousness of existence; in exercising his limbs; in basking in the sunshine, or cooling himself in the shade; and in the gratification of his mere animal propensities. But that such a primæval state of blissful ignorance, health and purity, ever existed, we have no other evidence than what rests on the fancies of poetry, or the dreams of poetic philosophy. The savages of the present day, who, one would think, ought to come most nearly to this blessed state of nature, present a picture the very opposite to that described. The tendency of man is obviously to civilisation and mental progress; whence the highest moral and intellectual advancement of which he is susceptible, is the only natural state that can be predicated of him. The mind, like the body, demands exercise. That the proudest faculties of our nature were intended for slothful inaction—that talents were given us to remain torpid and unproductive—is alike repugnant to reason and analogy. There is, in fact, no power of the living œconomy, however humble, but needs action, both on its own account, and on that of the general constitution. So closely united by sympathies are all our functions, that the judicious exercise of each one, beside conducing to its individual welfare, must contribute, in a greater or less degree, a healthful influence to every other.

It is, however, an opinion not uncommonly entertained, that studious habits, or intellectual pursuits, tend necessarily to injure the health, and abbreviate the term of life—that mental labours are ever prosecuted at the expense of the body, and must consequently hasten its decay. Such a result, however, is by no means essential, unless the labours be urged to an injudicious excess, when of course, as in all overstrained exertions, whether of body or mind, various prejudicial effects may be naturally anticipated. In support of this opinion abundant instances may be cited, both from ancient and modern times, of men eminently distinguished for the amount and profundity of their mental labours, who, being temperate and regular in their habits, have continued to enjoy firm health, and have attained a protracted existence.

The author, in the next place, considers the evil consequences that may be apprehended from overtasking the intellectual powers. He shews that the powers of the brain may be impaired by extravagant mental, in like manner as those of the muscles by severe corporeal, exertions. Hence, if the intellectual faculties are overstrained habitually, a train of moral and physical infirmities may be induced, which shall embitter existence, and abridge its duration. He then adduces reasons why intellectual operations should prove prejudicial more or less to health, viz. because such operations are necessarily more or less associated with passion—hence it is a matter of daily observation that those mental operations which elicit the strongest moral feelings are most prejudicial to the health.

We shall close our notice of this excellent and truly intellectual performance, not without urgently recommending its attentive and careful perusal to all who desire the *mens sana in corpore sano*.

---

### ON CERTAIN MORBID STATES OF THE LIVER. By Professer *C. H. Schultz*. Berlin.

*THE various states of the liver are intimately connected with the changes which may occur in its function. It is accordingly of great importance, to consider the hepatic function from an organico-natural point of view, so much the more, as this organ performs so important a part in the animal economy, both in the healthy and also in the morbid state. The function of the liver is closely connected with the portal system and the blood-*moulting*, nay the liver is to be regarded as the vertex or apex (die spitze) of the portal function itself. The liver has been frequently considered as a depuratory organ of the body, and the bile as an excrementitious substance; but this view is an uncertain and undefined one, because through it neither the direct relation of this depuration to the vitality of the blood, nor the organic end of the bile during digestion, have been correctly ascertained. The knowledge of the hepatic function is inseparable from the knowledge of the blood-renewal and its moulting; this function is related on the one hand only to the vitality of the blood, and acts on other organs merely indirectly through the blood; on the other hand, it acts through the secretion of bile forwards on chylification in the intestinal canal, and hence the liver possesses not only the importance of an excreting organ, but necessarily serves also as a key-stone for the periodicity of the blood's vitality between the commencement and termination of the same. The blood-moulting necessarily propels the biliary secretion; and the formation of new blood necessarily requires the bile in chylification. Thus the liver holds a middle place between the commencement and termination of blood-formation, and it may be acted on forwards and backwards in such a manner that certain morbid states may be occasioned thereby.

---

* As some of the terms and several of the principles which present themselves in this article, may be difficult to be understood by those not acquainted with the author's peculiar views in Physiology and Pathology, and as his character is very high in both these departments of science, we shall take this opportunity of giving a summary here, so far as may be necessary, to render this article easily understood. In the year 1842, Professor Schultz published a work entitled *Ueber die Verjüngung des menschlichen Lebens und die Mittel und Wege zur ihrer Kultur*—that is in plain English, *On the Youthification of Human Life, and the Ways and Means for its Culture.*

We are taught by physiology that the materials of which the body is composed are constantly undergoing changes, new matter being deposited and the old removed, or, as the author calls these two processes, the process of renewal and the process of separation, the effete or worn-out particles; to the latter process he gives the name of *moulting*. The substances which are moulted are but the *debris* left by the process of renewal. In another work of his, entitled *Systema der Circulation*, &c. the author has shown—that the blood-vesicles, called by others blood-corpuscles, are constantly undergoing renewal and disorganization—that the only organised constituents of the blood are the plasma or liquor sanguinis, and these vesicles—that the proper function of these vesicles is to absorb oxygen in the lungs—that their capacity to perform this function depends on their contractility—that the first origin of these vesicles is in the lymphatic system—that when this blood-vesicle is no longer capable of being acted on by oxygen from a loss of its contractility, it becomes effete, its colouring matter has to be removed, and itself has to be excreted—that it is in the liver all those changes are to take place—that it is in the vena portæ the old worn-out vesicles are taken out of the circulation, and the debris separated from the blood—that

1. First of all, the evacuation of the bile from the gall-bladder and the biliary ducts is affected sympathetically by the state of the intestinal motion. The intestinal motion is propagated sympathetically to the gall-ducts and gall-bladder, as may be readily seen by artificially irritating the intestine in an animal just killed, as in a duck, and on accelerating the intestinal motion the discharge of bile becomes also accelerated, and vice versâ, by retarding the intestinal motion, the motion of the gall-bladder, and of the bile-ducts are retarded and interrupted: a disposition to diarrhœa or to constipation acts in this way backwards on the liver, and the former will be followed by a hepatic flux, the latter by hepatic infarction, if these states should continue for any time. We find an instance of the first case, wherever there exists a disposition to the formation of acid in the intestinal canal of scrophulous children, or in the diarrhœa of adults occasioned by the same cause, and this state weakens the liver as well as the intestinal canal, so that at length the liver does not perfectly free the blood from the moulting material, and a disposition to jaundice is the consequence. In cases of hepatic obstruction, wherein a concentrated bile is contained for a long time in the gall-bladder, there arise dispositions to the formation of gall-stones, hepatic swellings, and hypertrophy, the moulting plastic material combining in such cases with the formative material, and thus producing increase of nutrition (des Vegetirens). In the case of the liver this is so much the more possible, as the vena portæ which contains the moulting blood exercises plastic powers similarly to arteries.

Another morbid state of the liver occurs in Summer and in Tropical Climates, whereby bilious fever, bilious vomiting, and bilious diarrhœas are produced. This state is occasioned by an excessive transformation of the melanotic blood into bilious blood immediately in the vena portæ. In order to understand this rightly, it must be considered that, for the secretion of bile in general, two sorts of conditions in the blood of the porta are necessary. 1st. The presence of a great quantity of colouring matter in the effete blood-vesicles, accordingly great quantities of black cruor, no longer capable of respiration and vitality. 2nd. That this colouring matter be no longer retained by the blood-vesicles, but be dissolved in the plasma whereby the plasma of the portal blood, as we have shown*, becomes redder, than arterial and venous blood. During its passage through the liver the portal blood in the healthy state loses the colouring matter dissolved in the plasma, which has been employed in the secretion of bile. The cause of this greater solution of colouring matter in the plasma of the portal blood lies partly in the greatly diminished contractility of the old bloodless membranes (of the blood-

---

these blood-vesicles are the true respiratory organs of the blood and subservient to the completion of the process of assimilation—that by absorption of oxygen the granular substance which constitutes the origin of the vesicle in the lymphatics is transformed into plasma, whilst the colouring matter is the residue of the transforming processes—that the plasma is the colourless organised plastic fluid in which the vesicles float—that this plasma is the true formative and nutritive material in the blood, and consequently that it exists in less quantity in venous than in arterial blood—that if the old vesicles are not excreted from the blood, as new ones form, the portal system becomes congested—that the last change the blood-vesicles undergo is *moulting*—that the blood purifies itself from these, and that the liver is the organ in which this exit from the circulation is made—that as the vascular system has no direct emunctory through which the moulted debris may be thrown off, the circulation through the liver pours them out into the intestines as bile—that the blood in the vena portæ differs from common venous blood in its colouring matter being darker, in containing less plasma, and in its plasma containing colouring matter, and in being more aqueous.—*Reviewer*.

* See the author's work entitled System der Circulation. S. 322.—*Rev.*

vesicles) which no longer retain the colouring matter, and partly in the greater proportion of water contained in the portal blood, whereby the colouring matter is more easily dissolved in the plasma.

We have now to consider, how far these circumstances are concerned in producing a disposition to bilious diseases in Summer, and in Tropical Regions. There is formed a great mass of bilious blood, the melanotic being changed into the bilious by solution of the colouring matter in the plasma. We have now to investigate the conditions under which the change takes place. They are chiefly two: 1. A greater debility and relaxation of the blood-vesicles, and a more watery state of the blood. These conditions are occasioned by the mode of life which men follow in Summer and in Tropical Regions: first, by the greater quantity of vegetable diet; and, secondly, by the greater quantity of watery drinks used.

1. Vegetable food forms in general only small blood-vesicles, and such as are provided with only very delicate membranes. This is seen in all graminivorous animals, which are found to have much smaller and more delicate blood-vesicles than carnivorous animals. Accordingly, a much greater quantity of water is required to dissolve the colouring matter from the blood of dogs and cats, than from the blood of rabbits and sheep. One may, with three or four parts of water, wash away all the colouring matter from the blood-vesicles of sheep and rabbits, whilst five or six parts, and even more, are necessary to wash it away from the blood of dogs and cats. Similar conditions are produced in the case of man by a preponderance of flesh or vegetable diet. To this it may be added, that the carnivorous blood-vesicles become much older, because they are more robust than the herbivorous. The herbivorous vesicles are, accordingly, much earlier in becoming effete, and in having this colouring matter dissolved, and hence it is evident how a mass of blood-vesicles which has been formed in man whilst feeding on a preponderating quantity of vegetable diet, especially raw fruits, which are eaten in such quantities in Summer, and in Tropical Regions, will evince such little resistance to the solution of its vesicles, so that there is already present a strong disposition to the formation of a red plasma, especially in the vena portæ. Hence it is, that vegetable blood, if I may so call it, disposes so much to liver and bilious affections. This is strikingly exemplified in the case of sheep, an extraordinary number of which die of liver diseases, so that scarcely an old sheep is to be found with a healthy liver. This is to be referred solely to the peculiar nature of the blood-renewal in these animals.

2. The attenuation of the blood by the excessive use of watery drinks, occasioned by the heat of Summer, and in Tropical Regions, forms the second cause of a disposition to hepatic disease. We have shewn elsewhere how by water-drinking from five to six per cent. of water, on an average, is introduced into the blood, and that this quantity more than suffices to dissolve so much colouring matter, that the entire plasma becomes reddened thereby. This effect is seen first in the portal blood, because the water drunk is absorbed from the stomach and intestinal canal by the roots of the vena portæ, (and but very little by the lymphatics,) so that the portal blood first comes in contact with the great quantity of water consumed, even though this same is subsequently diffused throughout the entire mass of blood. Now the more relaxed the state of the blood is, the less power is there to eliminate the water again through the skin or kidneys, and the longer will the water tarry in the blood, and exert its cruor-dissolving power in the entire mass of blood. Both these states now occur: the feeble relaxed state of the herbivorous vesicles, after the use of large quantities of vegetable food, and the excessive use of watery drinks: these are the necessary conditions for producing a disposition to bilious and hepatic diseases in Summer, and in Tropical Regions. In the next place, the liver becomes overloaded with bilious blood, the secretion of bile is exalted. Should this state continue, the bile secreted becomes abnormal in quality, and this circumstance produces disturbances of digestion. Again, this bilious state being transferred to the entire mass of blood, general febrile irritation arises, a disposition to jaundice, intermittent fevers, bilious fevers, which at length pass into nervous fever; whilst the arterial character of the blood is diminished in this state, the absorption of oxygen in the lungs is impaired, and accordingly the natural excitement of the nervous system is interrupted, its own healthy self-excitement being also destroyed.

That the liver itself should suffer most in consequence of these abnormal states of the blood-moulting, depends on this circumstance, that it is itself the normal blood-moulting organ, and that the morbid directions of this function press most upon it. The human liver is in Summer, and in Tropical Climates, reduced to the state of the sheep's liver, and, in fact, the study of liver disease in the sheep is well adapted to throw light on the diseases of the human liver. All these states are perfectly inexplicable, according to dynamical and chemical Physiology.—[*Beiträge zur Physiologischen und Pathologischen Chemie und Mikroscopiæ.* Berlin, 1844.]

---

Morbid Formation of Sugar as a Residuum of Abnormal Chylification, (not Abnormal Chymification.) By Professor *Schultz* of Berlin.

(Beiträge zur Physiologischen, und Pathologischen Chemie und Mikroscopie, &c. Berlin, 1844.)

The morbid formation of Sugar is most striking in diabetes; but sweet pus is often secreted in consumption also; and it is worthy of remark, that most diabetic patients die of consumption. No calculations have been hitherto made with respect to the quantity of Sugar voided in diabetes; but the quantity of sugar is of importance in reference to the qualities of the food, which become changed thereto. In diabetes, so much sugar forms in the urine, that of 62.03 solid parts in the same, 58.15 are sugar, and often still larger proportions. The fluid urine contains often from five to eight per cent. of sugar; so that in a discharge of five quarts of urine from eight to twelve ounces of sugar are voided. The sugar is grape-sugar, not always sweet, but still it has the property of fermenting. The blood of diabetic patients contains sugar only in small quantity, no doubt, as it is voided again. Rollo proved this to be the case; afterwards it was not believed; but Gregor, Bouchardat, and Simon have confirmed the matter. Gregor shewed that the sugar arises in the stomach, as it has been found in the vomitings of a diabetic patient, and even in the excrements. The latter is striking and interesting, as the formation of sugar in the stomach, especially from vegetable diet, is a general physiological phenomenon, which hitherto has been too much overlooked.

We have shown elsewhere, that all mealy aliments, and therefore bread, become changed into sugar by saliva. It may be an error, then, when Gregor states that he found no sugar in the gastric contents of a healthy man, premising that he had eaten bread or pulse with meat. But, according to Rollo, and according to our own observation also, diabetic patients form no sugar from meat, no more than the healthy stomach does. It has been shown that animal food is changed in the stomach only into lactic acid, not into sugar. The difference in diabetic and healthy cases consists only in this, that, in the healthy state, the sugar of the chyme formed from vegetable diet, becomes deoxidized again after the admixture of the bile in the duodenum, and, at the same time, is changed into an albumen-like substance; but this does not occur in diabetic states, so that here the sugar proceeds unchanged from the stomach into the intestine. *The cause of the morbid Saccharification in diabetes lies, accordingly, not in the Chymification-process of the Stomach, but in the Chylification-process of the duodenum,* and accordingly much more in the liver, which does not pour good bile into the duodenum for the purpose of a healthy chylification. Defective secretion of bile contains the grand cause of diabetes; hence, when there is a deficiency of bile, or when the bile is bad in quality, the intestinal contents contain sugar until its evacuation through the rectum. Besides all this, the chyme in the duodenum continues sweet and acid, during chylification it does not pass into the normal formation of albumen and fat, and in this lies the cause of diabetes.

We have made the observation, in the case of a diabetic female, that the medicinal use of fresh bile, from four to six ounces daily, perceptibly checks the formation of sugar in the urine. We have subsequently made several similar observations, more particularly in the case of a woman who was suffering under confirmed diabetes, and where, after the use of a sufficient quantity of fresh bile two hours after meals, the sugar entirely disappeared in the urine. The doses of bile hitherto given in diabetes have been too small; and hence, doubts have been entertained respecting its action.

The origin of the sugar accordingly proceeds from the intestinal canal, but not from the stomach, as M. Gregor maintains. We have twice found sugar in the lymph and portal blood of scrofulous children. But, true diabetes seems to occur only, when through the deficient formation of blood in general, a morbid process of waste has set in. For the sugar absorbed in the healthy state by the vena portæ is never eliminated through the urine, but, like all the nutritive parts of drinks, is deposited in the spleen and in the glands of the mesentery, and there subjected to a new elaboration and sanguification. Accordingly, the sugar introduced into the blood by nutrition, in the case of healthy dogs and horses, is not again found in the urine. The cause, why the sugar is eliminated through the kidneys in diabetes, a thing which does not occur in the healthy state, still requires investigation.

---

ON THE ORIGIN OF MELANOSIS. By *Professor Schultz,* of the University of Berlin. (Beiträge zur Physiologischen und Pathologischen Chemie und Mikroscopie, &c.)

IT has been the custom hitherto to represent melanotic masses as products of diseases of the liver and spleen; nor could it be otherwise, taking into consideration the hitherto state of our knowledge regarding the blood, and the share it takes in abdominal diseases. Heusinger has treated the subject at full length in his work on the spleen. But the truth is, both the liver and the spleen are wholly unconcerned in the formation of melanosis. According to what we have stated elsewhere, regarding the formation and development of the blood, and its two renewal-acts of formation and moulting,* melanoses arise solely from a morbid moulting of the blood. As the vena portæ is the natural rendezvous for the blood-moulting-material, the bile, disturbances of the blood-moulting will be usually connected with affections of the liver and spleen: but the spleen is just as much concerned in this as the glands of the mesentery, from which also the roots of the vena portæ take their rise; and the spleen is just as little primarily diseased as the liver, but merely in consequence of the moulting-ob-

* See the author's work, entitled *System der Circulation,* and *Verjüngung des Menschlichen Lebens.*—*Rev.*

structions of the blood. This is a point of high practical importance, as we thence see that melanosis is more an abnormal blood-moulting, thrown upon another organ, and depends on an overfilling of the vena portæ, and ultimately of the entire mass of blood with melanotic blood-vesicles, which may occur without any affection whatever of the liver and spleen. We have shown, on another occasion, that the cruor of the portal blood is quite distinguished from the cruor of the arterial and venous blood, and that, by its very dark colour, it closely resembles melanotic materials. That the separation of the melanotic masses is so frequently thrown on the lungs, was once considered as a proof that the lungs may take on the function of the liver, whilst the sole and only true cause is, that, amid the general melanotic state of the blood, this fluid becomes obstructed in the peripheric system of the lungs, and accumulates here, because it lacks the moving force, so that in the disposition to passive congestions of the lung, occasioned thereby, a tendency arises to throw off the melanotic materials directly in this organ.

With respect to the influence of the lung and liver in the so-called decarbonisation of the blood, an entirely incorrect view has been taken hitherto. It has been believed (and Tiedemann has taken great pains to prove it) that lung and liver may act vicariously in decarbonizing the blood, so that, when the function of the liver is interrupted, the lung takes on it that of the liver, and *vice versâ.* Tiedemann accordingly supposed, that throughout the entire animal kingdom there exists a contrast in the development of the lung and liver, that animals with large lungs have small livers, and animals with large livers have small lungs; as an instance of this, Tiedemann adduced Water-birds and Mollusca. But this is only apparent. The liver can only separate the black colouring-matter of the effete blood-vesicles, without ever having a necessity for oxygen, because the oxygen no longer acts on the defunct vesicles; its action refers only to the moulting-formation of the blood. The lung can only separate the carbonic acid from the young and still living vesicles, wherein the absorption of oxygen gas is necessary, in order to irritate the blood-vesicles to contraction; lung and liver never interfere with each other's function. In Mollusca the entire blood is found in a bilious state, because all the colouring matter is deposited in the plasma. These animals have therefore a natural black jaundice, and they are almost all black, and separate so much bile merely from this cause. A similar state of things is observed in water-birds without these animals having smaller lungs than other birds. It is, therefore, incorrect to maintain that the deposition of melanotic substances in the lungs depends on the natural tendency of the lungs to separate carbon instead of the liver. For the question here, is not concerning the separation of carbon in general, but concerning the separation of the black colouring matter of the blood. If the lungs deposit black-colouring matter in them, this is to be considered rather as a morbid state of the lung itself; a state in which the lung suffers only as a consequence of the action of the melanotic and of the bilious blood formed therefrom, and is thereby obliged to separate the black-colouring matter. Accordingly, it is not the lungs alone in which such melanotic substances are deposited; these depositions may take place in all organs, in the cellular tissue, in serous membranes, glands, in the brain, where obstructions of melanotic blood form, as is proved by experience. In Tropical climates European women sometimes have black menses. Nay, it is probable that the intestinal canal is much more frequently the seat of melanotic depositions than the lungs. After dysentery and yellow fever the entire intestinal canal is found to present as black an appearance as the biliary passages. It is certainly a circumstance worth noticing, that certain external influences which act on the lung, as air impregnated with marshy vapours, may produce a bilious state of the blood, wherein the lung then re-acts on the liver. But surely we must not infer from this a vicariousness in the action of the lung and liver; the real fact being that, in consequence of the carburetted-hydrogen gas, a paralysis of the blood-vesicles is produced, whereby much more colouring matter is dissolved in the plasma, and the bilious state is occasioned by the melanosis, which disposes to the secretion of the colouring matter abnormally, just as in the healthy state in the case of Mollusca. In all this the liver never takes on itself the function of the lung; nay, rather the liver still continues the portal of death, the lung the portal of life, with respect to the vitality of the blood. The lung must separate bile, and the liver must exhale carbonic acid, if these organs are mutually to interchange their respective functions. It is then much more to the abnormal state of the blood than to the action of the lung and liver that we must refer the formation of melanosis. The blood must throw off from itself the moulting-substance of the effete vesicles; the vascular intestine by which this is accomplished is the vena portæ. The melanotic blood is not attracted by the lungs; it is rather repelled by them; and it is attracted by the liver only in the healthy state, in order to eliminate the moulting-material as bile. If any obstruction occur here, the morbid deposition may be directed to various organs. If the liver become affected under these circumstances, it becomes so only in consequence of the abnormal blood-moulting, and not primarily; hence the liver is never to be blamed for the formation of melanosis, it is itself affected only when Melanosis has been formed.

---

### THE TRANSACTIONS OF THE PROVINCIAL MEDICAL AND SURGICAL ASSOCIATION. Volume XII. 1844.

THE present volume consists of TWO PARTS; the first part contains two retrospective addresses, one by Dr. T. Shapter, of Exeter, in which the improvements made in the various branches of medicine within the last year are presented to the notice of the Association; the other, by William Hey, Jun. Esq., Surgeon to the Leeds Infirmary, on the recent Improvements in Surgery. The Second Part consists of Essays and Cases. The first Essay is on *the Actual Process of Nutrition in the Living Structure, demonstrated by the Microscope; and the Renewal of the Tissues and the Secretions from the Blood thereby illustrated.* This is from the pen of William Addison, Esq., F.L.S., &c. already favorably known to the profession for his microscopical researches in Minute Anatomy. This Essay is accompanied with plates. We shall present to our readers a succinct analysis of the more important parts of this Essay. The author of the Essay first remarks that microscopical observers are not yet agreed with respect to the place of origin and the structure of the red corpuscles, together with the relation existing between them and the colourless ones. Both the red and the colourless corpuscles or cells circulate in all the capillary blood-channels of the human body. The object of the Essay is to trace the several stages through which *the latter* pass in their progress through the tissues, to describe the visible or physical characteristics of their contents, and to investigate the probable result of their final dissolution.

"The coagulation of the buffy coat of the blood," says the author, "is a *glaring instance* of an elastic, compact, fibrous, and colourless tissue, resulting from the fibrillation and incorporation of the colourless elements of the blood: the experiment related in the following paper is *a glaring instance* of the actual formation of this tissue, in which we see how the colourless blood-corpuscles and the molecules are included in, and incorporated with, the fibres; and, lastly, the accumulation of the colourless blood-corpuscles in the capillary vessels in the web of the frog's foot after immersion in warm water, their adhesion to the walls of the vessels, and their situation, first between the red current and the tissue, and then among the fibres of the latter, is a *glaring instance* of the process of nutrition."

Sect. 1.—Some years back the author published several cases demonstrating the existence of great numbers of colourless corpuscles in the buffy coat of the blood—he subsequently published the fact of the lymph globules being seen accumulating in the irritated vessels of the frog's foot; he then stated that a thin film of the coagulated fibrine from the surface of inflammatory blood had all the structural characteristics and physical properties of fibrous or membranous tissue. The process by which this tissue is formed he has called *fibrillation;* it takes place in the liquor sanguinis. These colourless corpuscles were found to exist in the blood at all times; and were more abundant in blood drawn from vessels whose calibre was increased by inflammation, or which were administered to an accelerated process of nutrition. The author, by following up his experiments, came to the conclusion that lymph and pus-globules, exudation-cells, and epithelium, originate from the colourless corpuscles. This conclusion was strengthened by the fact, that the fibrine of the liquor sanguinis was never seen during the progress of fibrillation to give origin to a corpuscle or globular particle of any kind. The author here details some experiments, proving that the buffy coat of the blood is neither more nor less than elastic fibrous tissue. By means of them he also endeavours to account for the formation of the buffy coat of the blood in all those cases in which its presence is connected with disordered function or disease. From sundry causes, the process of nutrition, or what amounts to the same thing, the function of secretion, is disturbed or diminished, and the colourless corpuscles, therefore, accumulate in the blood. When a vein is opened, a greater number than usual flow out; from the sudden change of temperature to which they are exposed, or from other causes, many of them burst or become ruptured, and discharge their contents, consisting of a liquor sanguinis and molecules. After standing a short time, the liquor sanguinis rises to the surface, drawing up not only the molecules which were associated with it in the interior of the corpuscles, but also all those colourless corpuscles which have preserved their integrity amid the changes to which they have been exposed; here it very shortly fibrillates and forms tissue, as it would have done in the living vessels, had the contents of the corpuscles been appropriated to the purposes of nutrition. Hence, neither the fibrinous element nor the serum circulate in the blood as part of the fluid in which the red corpuscles are suspended in the living vessels; they are both inclosed within, and form, incorporated together, an essential ingredient of the interior contents of the colourless corpuscle. He ascertained by experiment, that on placing a drop of white and opaque healthy pus on a slip of glass, and well mingling it with a drop of liquor potassæ, it entirely lost its opaque character, and became clear and transparent, resembling mucus—the effect of the alkali is to destroy the pus-globules. This experiment sets at rest the difference between pus and mucus, and confirms the identity of mucus and pus-globules. From a number of experiments here detailed the author draws the following conclusions :—

1st. That the plastic fibrillating liquid, denominated liquor sanguinis, exists as a fluid within the colourless blood-corpuscles, and that, when it escapes from them, it forms an elastic fibrous tissue, the serum being the residual liquid.

2nd. That mucous and pus globules are altered colourless blood-corpuscles, and that the glairy fluid termed mucus is nothing more than an altered state of the fibrillating liquor sanguinis, the change from the one to the other being coeval with the changes which characterize the microscopical aspect of the corpuscles. Hence, if we take the red portion of the buffy clot, and the red blood-corpuscle, to represent blood, then the colourless layer of liquor sanguinis with the colourless blood-corpuscle will represent the first remove from blood, and mucus or pus, with the mucous or pus globule, will be the next. And it would appear, generally, that the nearer the corpuscle is to, or the fewer the stages of its removal from, the circulating fluid, the more nearly it resembles the colourless blood-corpuscle, and the more decidedly and visibly its fluid contents, when they escape, fibrillate; whereas, the further the corpuscle is, or the greater the number of stages of its removal from the circulation, the larger it is, the more it is filled with molecules, and the less perfectly do the fluid contents fibrillate. Now, if it be admitted, that the fibrillating liquor sanguinis is changed into mucus in the interior of living cells, then there can be no difficulty in admitting that similar living cells, by a different mode of elaboration, may form not only sundry kinds of fibrous or mucous tissue, but the tears, saliva, milk, or bile.

His subsequent investigations have only tended to confirm the conclusions drawn by the author from his previous "Researches," viz. that the colourless corpuscles are very highly organized cells, within which the special tissues, and the secretions are elaborated; and it appears that the renovation of these tissues and secretions from the blood does not take place by the cells discharging their contents into the general mass of the circulating current, to be separated therefrom by some peculiar transcendental, and purely hypothetical selective process of exudation, through a structureless and transparent tissue, but, by being themselves attached to, incorporated with, and performing their special function in the structure.

The author is satisfied, from the facts upon which the above conclusions are founded, that the progress of the colourless blood-cells in administering to the maintenance of the living body is not *back again* into red corpuscles, but *onward* into the higher forms of organised fibrous tissue and epithelium. He conceives also that the blood-cells have their origin in the chyle, and that every step, by which they multiply or increase in numbers, is progressive, carrying them higher in the scale of organization, until, at length, they acquire colour by passing through the capillaries of the lungs: they then again become colourless by further elaboration or nutritive changes, and in this condition are prepared to enter upon what may appropriately be termed a higher and more special state of existence, *i. e.* into the composition of the tissue. If, then, the colourless blood-corpuscles be termed "parent cells," they must be considered as pregnant with the embryo materials of the tissues and secretions, and not with "young blood-cells." According to this view of the entire dependence of secretion upon the nutritive process, neither milk, mucus, nor the bile, are derived from the blood as such; on the contrary, they are each elaborated in the interior of cells, which were previously colourless blood-cells, the change from a blood to an epithelial cell being the sum of the process of secretion; and milk, mucus and bile, are the visible fluid results of the final dissolution of the cells. Hence, then, a secretion is the result of the last stage of the process of nutrition, and hence, also, it is by the special vital activity of individual cells, and of all the visible particles composing their structure, that the secretions are produced.

SECT. 2.—The author considers that the hitherto-received doctrine, viz. that the capillary blood-vessels have permanent tubular coats, is totally irreconcileable with the results of recent observations regarding the function and process of nutrition. He gives the following explanation and *theory of the process of nutrition:*

Nutrition may be normal or abnormal. In normal nutrition the colourless blood-corpuscles adhere to the tissue forming the boundary of the blood-channels; they contribute to form this tissue, *i. e.* the parietes of the capillaries, and are afterwards evolved or thrown off from the nearest free surface—a follicle, crypt, or duct, constituting epithelial cells; the mucus, or the secretion given out by the follicles or flowing through the ducts, being the result of the final dissolution of the cells and tissues. In abnormal nutrition the colourless blood-corpuscles adhere to the parietes of the capillaries in much greater abundance; they pass out among the fibres of the tissue, which is now much less fibrous and coherent, and are subsequently thrown off from the nearest free surface—a pyogenic surface, as lymph, pus, exudation, or imperfect epithelial cells.

According to this account, the fibrous walls of the capillary vessels are formed by the fibrillation of the liquor sanguinis contained in the interior of the colourless blood-corpuscles, some of the corpuscles being employed or expended in forming the fibrous tissue, others passing through it in the interstices of the fibres for further elaboration into epithelium. If the corpuscles congregate in unusual numbers, and be hurried through the stages of their growth by an abnormal nutrition, they come under observation as lymph, pus, or exudation cells; the various appearances presented by these objects, and the quality of their contents, being referrible to the period of their growth, to the special function of the tissue, and to the chemical action of the fluids with which they are associated.

The author sets about testing the truth of this, his theory of nutrition, by referring to the phenomena of some well-marked disease. He takes for example scarlet fever: this disease has a specific character arising from a peculiar poison affecting the blood-corpuscles; the function of nutrition becomes more or less disturbed, the secretions are diminished or disordered, and the colourless blood-cells consequently accumulated in the circulating fluid. When the disease is mild, these cells are determined to the epithelial surfaces of the skin, in order to be eliminated from the body. In the severer cases they accumulate in the vessels of some of the internal epithelial surfaces, causing muco-purulent discharges, or the formation of abscess, so that there may be scarlet fever without cutaneous redness. If there has been no exfoliation of the skin, nor critical discharge, the abnormal corpuscles either have not been at all, or only imperfectly eliminated. Under such circumstances, the patient may appear for a little time to be getting well; this is deceptive, as the diseased corpuscles are still in the circulation, insidiously perhaps, by an abnormal nutrition, locating themselves in some glandular structure, or forming tubercles on the lungs—if not so, their presence will be speedily proclaimed by symptoms of dropsy, or some acute inflammatory or abnormal nutrition in the kidneys, the phenomena of which attest the truth of the theory; for the disintegrated tissues and the red corpuscles of the blood are voided in the urine.

These views explain why it so often happens that the mildest cases are so frequently followed with graver lesions after—it also points out the necessity of attending to the exfoliation of the cuticle or of watching some other critical discharge before dismissing the case as one which has terminated satisfactorily. To test the theory still further: the author asks, what are the remedial measures it suggests, where the natural cure—the elimination of the abnormal corpuscles—has been interrupted or impeded?

1st. Bleeding, in order to diminish the total amount of the colourless blood-corpuscles; and by so doing, to recall into the current of the circulation, some of those which have become stationary, oppressing and loading the tissues.

2. A more or less active purgation or accelerated nutrition upon all the available epithelial surfaces, so that by removing the effete epithelium, a stimulus may be given for the more rapid growth of the new. Thus then the theory of nutrition advanced by Mr. Addison suggests the employment of those remedial measures which enlightened medical experience has sanctioned as frequently essential to the cure of the disease. Nay, it even does more; it indicates two very different modes of withdrawing blood—one by lancet, leeches, &c. where both the red and colourless corpuscles are discharged; the other, by establishing an accelerated nutrition—an eruption on the skin, a blister, an issue, or seton, where the colourless corpuscles only are selected and thrown off.

The 3rd Section of this essay treats of the *Function of Structure*; the author's object in it, is to point out those analogies and facts most calculated to give an insight into the means whereby the qualities or properties of the living structure are sustained by a succession of altering cells. The general tenor of the argument, several of the illustrations, and some of the conclusions arrived at, are identical with those employed by Liebig in his Organic Chemistry. There is, however, this difference. Liebig proceeds on the supposition that the globules of the blood take no share in the process of nutrition, whilst our author assumes that the globules of the blood are the sole agents of nutrition. In this section the author assumes, as a leading and essential principle, "the inherent activity of matter"—this he proves from authority; first referring to Sir J. Herschel, who says that the inherent activity of matter is proved not only by the production of motion, by mutual attractions and repulsions of distant or contiguous masses, but by the changes and apparent transformations, which different substances undergo in their sensible qualities.*

Bacon says, that "all bodies whatsoever, though they have no sense, yet they have *perception.*" As the ultimate elements of all living bodies are of the same kind as those most energetic in inanimate and inorganic bodies, so they have the same "inherent activity;" and, being in a constant and ceaseless state of change, it follows (unless the contrary be proved) that a certain amount of power or force must be the result. Also, as the secreting and all the nutritive organs of the body are structural arrangements, in which changes are going on, they can scarcely be supposed limited in their office merely to the production of what is termed a secretion; on the contrary, there being a large amount of "perception," or "susceptibility" and power, quite distinct from consciousness and volition, to account for, it is much more probable that their function is a *dynamic function,* and that the secretions flowing from them are the visible remains of the materials which have ministered to the function. After considering the will as another of the sources of motions in living bodies, of those motions which are called voluntary, he lays down this rule: that all the visible motions and affections of the body distinct from the *will,* may generally be modified or removed by altering, increasing, or diminishing the nutritive changes of the structure: and it may be affirmed generally that the will, having a large control over the materials received within the body, and therefore over the nutritive changes, is responsible for the origin of many bodily diseases. The metaphysical nature of the disquisitions constituting this section obliges us to discontinue further notice of it.

---

* Preliminary Discourse, p. 297; also pp. 59.

